# Bibliology; or Analytical Reviews of Medical Publications

**Published:** 1819-01-01

**Authors:** 


### Second Department.

### BIBLIOLOGY;

OR

### ANALYTICAL REVIEWS OF MEDICAL PUBLICATIONS.

Nec tibi quid liceat sed quid fecisse decebit
Occurrat, mentemque domat respectus honesti.

CLAUD.

### Medicine.

### I.

*Practical Observations on the Action of Morbid Sympathies, as included in the Pathology of certain Diseases; in a Series of Letters to his Son, on his leaving the University of Edinburgh, in the Year* 1809. By ANDREW WILSON, M. D. Kelso. One vol. Octavo, pp. 407. London, 1818.

IN various parts of this Journal, but particularly in our review of Dr. Parry's Elements of Pathology, we took occasion to protest against the present rage for tracing diseases almost exclusively to *vascular* derangement, and overlooking the morbid movements or state of the *nervous* system. The longer we live, the more we see, and the deeper we study, so much the more are we convinced, that, not only are the *primary impressions* of morbific causes sustained by the *sentient* system of the human fabric, but that it is in *this* system the primary morbid *movements* first begin, and are thence propagated to the *vascular* system; which, from that moment, reacts upon, and is again influenced by, the nevous system. We never shall have a sound pathology or successful practice, while any one of these two systems is studied in preference to the other; or, while they are considered as unlinked in perpetual reciprocity of action. Under this conviction, we shall give every possible publicity to the labours of those observers of Nature, who are exploring this interesting subject; and we could not have a better opportunity than in the analysis of Dr. Wilson's volume—a work which is characterized by deep thought, solid judgment, and accurate observation.

Dr. W. justly observes, that the laws of morbid sympathies are often very demonstrable to the senses, "and may be reasoned on in diagnostics with the utmost propriety, and considered as steady principles to be acted upon." Page 7.

"We know, for instance, that certain irritating matter contained in the stomach, will very commonly produce a head-ache; but knowing also the universal influence which the stomach exercises over the whole human frame, if a person becomes affected with pain, &c. in a distant part of the body, in a limb for instance, without any occasional cause having been applied, attended at the same time with certain signs of a loaded stomach, reasoning from the above facts, we readily believe, that if irritation in the stomach can produce pain in the forehead, it may, at any other time, produce pain in any other distant part also. It is a fair enough inference, therefore, that in this case likewise, the primary disease is seated in the stomach, and is a cause of pain in the limb by an act of morbid sympathy." Page 8.

The brain and its appendages, indeed, being the medium through which the living principle acts upon the otherwise inert organic structure, as also the medium through which the mind is made conscious of injurious applications offered to any part of the machine, it may properly be said that every part of the human body is capable of sympathising with the rest; because we see that no part of it can admit of having the sense of feeling excited to the height of pain, without the whole frame being deranged in sensibility.+ There are however specific sympathies, which are so common in their occurrence, as to be noticed as things of course; as, for instance, the gastro-cerebral, the cutaneo-gastric, and the utero-gastric sympathies.

These morbid sympathetic impressions on distant organs or parts are sometimes attended with pain, and sometimes not. In the *latter* case, the original irritation only increases or decreases the excitement in the sympathising structure. Thus we see the *vascular* system sometimes thrown into *inordinate* action by acrimonious irritation in the aliamentary canal; at other times, the action of the heart is almost suspended by the application of offensive substances to the nerves of the stomach. But when the sympathetic irritation arises to the degree of *spasm*, which, in fact, is only the highest degree of contractile action, in a muscular or fibrous structure, then we have *pain*.

"Wherever, therefore, muscular fibres exist, morbid spasm may also exist; consequently, not only the fibres of large muscles, but the smallest fibrous threads of all the membranous parts, are

See M.S. note also susceptible of spasm from irritation, applied either directly or indirectly from morbid sympathy.

"It is further to be observed, that although the most violent spasms of large muscles are seldom, if ever, succeeded by local inflammation, as is instanced in epilepsy, hysteria, &c.; yet, when the muscular texture of a membrane becomes the seat of a sympathetic spasm, *local inflammation of that membrane is speedily induced, the coats of its capillary vessels being probably also soon included in the sympathetic influence.*" Page 22.

This is an important doctrine, which is ever to be borne in mind by those who separate spasm from inflammation, and never dream of the *latter* when they have to combat the former.

With regard to the vinculum, as Dr. W. justly observes, connecting the sympathising part with that where the irritation is applied, we remain totally in the dark; and therefore, all we have in our power on this point is, by careful observation, to mark demonstrable facts; and these, by candid induction, ought to lead to correct practice.

The extensive influence which a healthy or diseased state of the digestive organs, and of the stomach in particular, exercises over the whole human frame, mental and corporeal, has long been, and still continues to be, the subject of observation and wonder among pathologists. When, indeed, we consider that the mucous membrane, stretching from the mouth to the anus, and lining so many important hollow viscera, is a secreting surface, studded with innumerable nervous or sentient papillæ, and exposed daily to a mass of heterogeneous and stimulating matters, as well what is poured in, in the shape of food and drink, as poured forth from the mucous glands themselves, in the shape of morbid secretion, it is not to be wondered at that the primæ viæ are the seat of most of those primary irritations which are productive of disease. The morbid sympathies which subsist between the stomach and various distant parts are doubtless reciprocal; but those which radiate *from* the gastric centre, would appear to be more powerful than those which are propagated from distant parts to it. It must also be remembered, as was hinted before, that irritation in the fibrous structures of the membranes lining the hollow vicera, and entering into the composition of joints, &c. will induce first spasmodic action; and whenever *this* arrives at a certain height, inflammation in the structures under consideration. This local inflammatory movement will soon draw the heart and vascular system into increased action, by morbid sympathy—and thus a febrile state is induced The following passage from Dr. Wilson depicts precisely our own sentiments, and accords with our own practice and experience.

In fact, the operation of the *laws of sympathy*, through the nervous influence on the living system, is a thing demonstrable to the senses: and any theories which I have ventured to offer on their pathology, are very nearly as much so, to an unbiassed mind. At least, this I will assert for myself, that to my conception, they approach far nearer to the certainty of demonstration, than any other pathology I have met with on the same subjects, and have served to *direct my own practice*, in a satisfactory manner, through a mist of insulated facts and general assertions; while every additional year's experience has served more and more to strengthen my opinions." Page 36.

We believe that Darwin, the great father of the sympathetic doctrines of modern times, was the most *successful* practitioner that England or the world ever produced. There was scarcely a town on the Continent of Europe from whence he was not consulted, either personally or by letter; and we have it from good authority, that his success was mainly owing to the *multiplied resources* which the sympathetic views of disease opened out to him. In our own practice, for many years past, we have had *solid* reasons for pursuing the doctrines and practices of Darwin; and we seriously recommend them to the rising generation, who are not yet so blinded by prejudice or pride, as to be beyond the reception of any thing in the shape of advice from others.

In the fourth Letter, our author enters on the consideration of febrile diseases. He considers that "a permanently increased action of the heart and arteries" is a *sine qua non* in fever, no other phenomenon being invariably present. We think, however, that a *deranged action* of the heart and arteries is a more permanent symptom in fever than an *increased* action; but we shall not cavil about definitions. It is of more consequence to bear in mind that this increased [deranged] action of the heart and arteries may arise

"From irritation, applied to the nerves of some distant part of the body, between which and the vascular system a primary affinity subsists, exciting that system into violent action by morbid sympathy; or from the effects of something of an irritating nature having been received into, and circulating with the blood, and thereby directly applied to the internal surface of the heart and blood-vessels themselves. But the establishment of a febrile state of the system, as derived from remote causes within the body, appears very demonstrably to acknowledge the following primary distinctions:

" 1st. As derived from the effects of acrid matter applied to some department of the nervous system, and exciting a morbid action of the heart and arteries by morbid sympathy; but such a remote cause, experience shows, is most commonly seated in the digestive organ.

" 2d. As derived from the effects of irritation, arising from organic obstruction, somewhere existing in the body, and exciting increased action of the heart and arteries, by morbid sympathy.

" 3d. As dervied from the effects of something of an irritating nature received into, and circulated with the blood.

" 4th. As derived from passions of the mind, exciting an increased action of the vascular system, by morbid sympathy.

" 5th. As derived from two or more of these primary causes of fever acting at the same time." Page 43.

Our author candidly acknowledges that we are totally ignorant why gastric irritation should in one person excite the vascular system, and in another the voluntary muscular system; but we are certain that such is the fact. Dr. W. then proceeds to the consideration of gastric fever, as the most common, and at the same time, the most simple form of fever. He considers the irritation in the stomach as propagated, by morbid sympathy, to the heart and arteries. In this species of fever, the first signs of any departure from health are of that kind which point out impaired action and morbid irritation in the digestive organs, as is evinced by the precursory phenomena of diminished appetite; weight at the præcordia; abdominal distention; thirst; parched mouth, and furred tongue. To these are commonly added, signs of impaired and irregular action in the nervous system, arising from morbid sympathies with the primæ viæ; such as languor, disturbed sleep, head-ache, dry skin, flushings and chills. This is the prevailing fever of infantile years; and, if taken in time, may soon be checked, merely by the ejection of the source of irritation from the primæ viæ; another proof of the truth of the doctrine in question.

The two great indications then in gastric fever are, the evacuation of the morbid secretions from the line of the alimentary canal, and the reduction of morbid temperature on the surface. Dr. W. here takes occasion to object to the unreasonable prejudice which has lately existed against emetics in this country, chiefly engendered, we conceive, by their deleterious effects in the hotter regions of the earth, where the gastric and biliary systems are ordinarily in a state of irritability, which is greatly exasperated in the fevers of those climates, and of course where emetics are improper. But in this country it is very different, and physicians lose a powerful febrifuge by the disuse of emetic tartar in particular.

"Nor are antimonial emetics attended by any hazard, nor even by more severe nausea than ipecacuan, unless it happens by an over-dose, or by mismanagement during its operation. One grain of ant. tart. for an adult, will generally nauseate the stomach in half an hour; when, without waiting longer, the stomach should be distended by drinking warm water, and vomiting is promoted by irritating the fauces a little with a feather. One half-glass of wine after every fit of vomiting will assists its operation." "It is by a prudent and free administration, then, of antimonial emetics, along with mercurial laxatives, assisted by the neutral salts, that the first and great indication of cure is here to be accomplished." Page 60.

The Doctor takes this opportunity of expressing his opinion that the *debility* in these fevers is only apparent, not real; and of course, to be removed or prevented by evacuations, which take off the load from the spring of life. He justly remarks, that mere *aperients* will not do; but that "very free evacuations are always necessary, procured by the help of the most active *purgatives*, administered in appropriate doses; remarking the nature of what comes off, till it puts on a healthy appearance, a circumstance which usually attends a solution of the fever, with its accompanying train of symptoms." Page 62.

Our author next turns to the consideration of fever, as dependent on "idiopathic organic obstruction;" viz. topical inflammations, from whatever occasional source they may be derived as local stimuli, wounds, bruises, &c.

"The application of excessive cold, or of burning heat, &c. forming obstructions in the capillary vessels of the vascular system, and exhibiting what is designed local inflammation; from whence, by a sympathetic affinity, the heart and arteries are drawn into violent action, and it thereby becomes a primary cause of fever." P. 71.

After some ingenious remarks on the nature of the topical inflammation itself, in which he concludes that the obstruction to the free stream of blood is seated in the capillary extremities of the veins, our author observes, that with respect to the *treatment* of fever in these cases,—

"Large and general bleedings, as also topical bleeding whenever it can be applied, are perhaps the most powerful means; and along with these, aperient medicines. But, whenever an acting morbid cause is observed to exist in the digestive organs, a free discharge from these, *by the most active evacuants*, is a measure of great importance, from the mutual sympathy betwixt the stomach and every part of the body. As to external applications, I have always observed tepid fomentations to be the most useful, and the most soothing to the patient's feelings." Page 80.

The ratio symptomatum, and pathology of typhus are next descanted on by our author, with much ingenuity and acuteness; and although we cannot agree with him on all points here, particularly in his opinion that the gastric and biliary secretions are greatly *increased* at the beginning of typhus, yet his doctrines all tend to support the propriety of the depletory or anti-stimulant plan of treatment now superseding the Brunonian mania. Dr. W. believes, and we unite our suffrage to his, that cinchona stops the paroxysms of an ague, not through the medium of the circulation, but by its tonic, and perhaps sedative effects on the nerves of the stomach. Indeed, it is only in this way that the salutary operation of several other remedies in intermittent fever can be accounted for.

In respect to our author's *methodus medendi*, we think it is not quite energetic enough; though, probably, in the part where he resides, and where fevers are not likely to assume the graver types, the practice may be good enough.

Dr. Wilson's long chain of reasoning on influenza we must pass over, in order to pay some attention to his opinions on acute rheumatism. Here the sceptics of the day will be ready to say, that Dr. W. far outstrips our illustrious London pathologist, Mr. Abernethy, in attributing a variety of diseases to derangement of the *digestive organs*. Although we are not disposed to go the lengths of either Dr. W. or Mr. Abernethy, on this subject; yet, we are convinced, that there is much truth and justice in the doctrines of both these gentlemen.

"Acute rheumatism (says Dr. W.), in its progress, appears to include two stages of disease; the first of which is *gastric fever*, attended by a painful state of the membranes surrounding certain joints, excited by a spasmodic affection fixed on them, in consequence of morbid sympathy, with irritation in the primæ viæ. The second stage is formed by an inflamed state of these membranes, created by the continued action of the same sympathetic affinity; which inflammation is occasionally so considerable as to produce effusions in the cellular membrane, and even the bones of the articulation become sometimes inflamed, eventually giving rise to anchylosis, and other diseases of the joints." P. 221.

On these principles, Dr. Wilson employs liberal evacuations from the primæ viæ, especially by calomel and antimony, aided occasionally by the neutral salts, particularly the tartrite of potass, or phosphate of soda, "till the signs of a loaded state of the digestive organs shall disappear, or at least, shall have greatly abated. Anodynes, which have the effect of suspending spasm, and of procuring a state of rest, are useful from the beginning; but their usefulness is most eminent after the primary irritation is lessened," P. 223. +

Our author employs bark and steel, when the febrile state is subsided. For the local inflammation he trusts principally to topical bleedings and fomentations, "a little more than blood warm." He also recommends warm anodyne poultices, composed of the common forms, with opium or cicuta added. He is an advocate for calomel and opium in acute rheumatism.

From this disease, the transition to gout is easy. Our author not only supports the idea of a strong affinity between gout and rheumatism, but comes to the conclusion that both diseases mainly depend on derangement in the digestive organs.

"In contemplating the above premises, it is obvious that there appears no other demonstrable cause of vascular excitement, but that which, by unequivocal signs, is shown to exist in the digestive organs; therefore, this unavoidable conclusion is likewise obvious, that the febrile paroxysm excited in the attack of a fit of gout must be viewed as real gastric fever; and farther, that any other suppositious principle, as inherent in the system, but which is undemonstrable as a primary agent, or remote cause of the disease, may fairly be esteemed as imaginary.

This doctrine, indeed, is very little different from that inculcated in some of the latest and best works on gout. In respect to the treatment, Dr. W. justly observes, that over the predisposition or idiosyncrasy of the patient, medicine has little control; yet, many of the inordinate movements in gout may be soothed and moderated—"especially by evacuations from the stomach and bowels."—Dr. W. considers also, that such medicines as act upon the skin, are extremely beneficial; as the liq. ant. tart. and tinct. opii, accompanied with diluting drinks. The acetate of ammonia also, given in tepid drinks, is useful for the same purpose. In respect to local applications, "the tepid warmth of an emollient poultice, void of every kind of acrimony, is the most suitable; or, tepid emollient fomentations, covering the parts afterwards with warm flannel. To these may be added, topical bleeding with leeches." P. 334.

In the intervals of the paroxysms, Dr. W. recommends the greatest attention to the state of the stomach and bowels, particularly by laxative medicines.

+ Dr W. was probably unacquainted with the virtues of Colchicum, at the time he wrote. Feb. 1830

"Of the last, calomel is the most efficient, and ought to form the chief basis of the measures taken for clearing the lower intestines, accompanied with the occasional use of some saline purge."

Emetics, he thinks, are valuable remedies in the remissions of the disease. He condemns the external application of cold, lest "some other organs, which may be next in sympathetic affinity with the seat of irritation, become affected with its influence; and if this shall happen to be any vital organ, the result may be fatal." P. 339.

Dr. W. next proceeds to draw a parallel between gout and erysipelas—a parallel which has been very fully drawn by the continental physicians, as lately translated by the Editor of this Journal. We shall quote the following case: omit

"A gentleman has had an ulcer on his leg for many years. Along with the ulcerated leg, he is, at the same time, subject to bilious accumulations in the primæ viæ; but these accumulations seldom occur without producing a febrile paroxysm, attended by extensive erysipelas around the ulcer; under the influence of which he has occasionally laboured for two weeks at a time, but without any medical aid, further than what he derived from some gentle aperient medicine to preserve his bowels soft. At last, when under one of these feverish fits, he was persuaded to take, though reluctantly, an antimonial emetic, and even to repeat it, followed with a mercurial laxative; by which means he threw off a great quantity of offensive bilious fluid, after which the inflammation and fever very quickly abated. Since this time, as the ulcer on his leg still remains, and continuing still subject to bilious complaints, he has become aware of the approaching illness; and being now convinced of the benefit attending this practice, he has, by seasonable evacuation, saved himself from many a fit of fever and erysipelas." P. 376.

Dr. W. observes, and wisely observes, that irritating matters, and consequently irritation, cannot be expected to be removed from the long line of the digestive organs by a few evacuations, whether of the emetic or cathartic class. Part of the offending matter will often lurk in the duodenum, or some of the sinuosities of the other intestines, while the ordinary discharges by the rectum are suffered to pass along. Additional morbid secretions may also be collected anew— omit/

"Therefore the evacuations ought to be persevered in till the signs of irritation in the stomach shall disappear, and till the discharges from the rectum shall assume a healthy aspect; otherwise, the portion of vitiated matter which is retained, will assuredly tend to prolong the disease, and disappoint expectations." P. 379.

*Angina pectoris.* Dr. Wilson thinks that post mortem researches "have offered no certain information, nor discovered any organic disease within the thorax, which might be referred to as the direct cause of its symptoms." 382.—Here we cannot coincide with our author. The Histories and Dissections of Dr. Parry, and cases which have since been published, afford very satisfactory evidence that all the symptoms of angina pectoris have *sometimes* resulted from ossification of the coronary arteries. We are ready to admit, however, that all the phenomena of syncope anginosa have *also* occurred, and terminated even in death, without any disease of structure being cognizable to the senses. This must lead all unbiassed minds to the conclusion that it is sometimes a disease of structure, giving rise to periodical lesion of function—and at others, only a derangement of *function* in the respiratory and circulating organs, leaving no perceptible trace of its previous existence after the thread of life is snapped In the latter cases, we can only suppose it to be of a spasmodic nature, resulting from arthritic or rheumatic irritation misplaced, and not yet amounting to ostensible organic change in any tissue or vessel. It is in this light that our author views it, and we shall present his ideas on the subject.

> "In the course of my own experience, I have seen but a few cases of this kind; but from my observations on these few, I have uniformly discovered, that at the commencement of the disease, the digestive organs were in a loaded state; as also, from farther inquiry, that they had previously been so. But from considering the strong disposition to sympathetic action which subsists, in some individuals, betwixt these organs and the heart, as well as other organs within the thorax, together with a general review of the predominant symptoms, I cannot avoid drawing this inference, that angina pectoris is a disease which also arises from a morbid sympathy, with a primary cause, seated in the primæ viæ. P. 385.

With respect to the particular seat of the spasm, Dr. W. thinks, that it is doubtless either in the heart itself, or the adjoining great blood-vessels, or perhaps in both at the same time; he also thinks that from the fixed severe pain under the sternum, a little to the left side, and at times, quite across to the back, the mediastinum may be very materially included in the spasmodic affection, perhaps in a primary manner, as in arthritic or rheumatic translations. It is certain, indeed, that angina pectoris chiefly attacks those of a gouty or rheumatic character. We shall introduce an interesting case in elucidation.

" April 18th, 1777. Mrs. ——, aged 50 years, complains of an intolerably severe stricture extending quite across her breast, attended by a severe pain under the middle of the sternum, which communicates itself to both her arms in a violent manner, but confined to a spot about the middle of the humerus; accompanied with a feeling of suffocation, and a most irregular, soft, intermitting pulse. In this state she continues for the space of ten or fifteen minutes, when the symptoms gradually abate, and she feels free from any complaint, excepting weakness, her spirits being much exhausted. Her appetite for food is impaired; her tongue much furred, with thirst; bad taste in her mouth; weight at the præcordia, and restless nights; her pulse, excepting when under the fit, being natural. She is of a spare habit, and subject to atonic rheumatism in her arms and limbs, and has been repeatedly affected with violent periodical head-aches; she has been complaining in this way for some days. At first, the fits returned twice or thrice in the twenty-four hours, with unequal violence; but she feels them increasing in frequency as well as in violence, and now a recumbent posture is certain of producing them at any time.

" She had, on some former occasion, been alarmed by the severe operation of an emetic, and I found, at present, that her aversion to it could not, as yet, be conquered. I was on that account left to the effect of laxatives alone, for the purpose of clearing out her stomach and bowels. These were accompanied with opiates, and a blister on her breast. The first cathartic dose produced some bilious stools; and a repetition of it every morning, with a powerful opiate three times daily, had the effect of mitigating the violence of the disease for a few days; after which it returned with increased violence, both in length and frequency of the fits, accompanied with faintness; her pulse was extremely confused, and she described her feelings of anguish as intolerable. She had now been out of bed for eight nights and days, because any attempt at a reclining posture was certain of producing a paroxysm; on which account she was compelled to spend the whole of her time in her chair, and, notwithstanding the opiates, she slept very little. I now at last prevailed on her to use an emetic of ant. tart. and she discharged a great quantity of acrid, bilious-coloured fluid. On the succeeding night she enjoyed more sleep, but still could not bear a lying posture. On the 27th the fits less severe, and less frequent; she continues the opiates; and on the 28th took a laxative dose. On the 29th relapsed, and took another emetic; threw up much bile, and was again relieved.

" May 2d, took another emetic yesterday, and has had no fit for twenty-four hours—can sleep in bed. May 4th, has had some slight returns yesterday, and repeated the emetic; discharged more offensive bilious fluid, and is better. May 10th, continues better; her tongue is clean, and other signs of a loaded stomach, &c. gone off. May 29th, has taken stomachic bitters, with the daily use of aperient pills; and by the help of moderate exercise, and restorative diet, remains quite well. She had afterwards repeated slighter attacks of the same complaints, which were always removed by a discharge of offensive matter from the intestinal canal, procured by the same means, and died at last of another disease in the year 1790." P. 390.

Finally, our author is convinced, from a careful attention to the phenomena of croup, that the primary seat of irritation is in the digestive organs, whence is generated, by morbid sympathy, a spasmodic affection of the glottis, ending in inflammation of the mucous membrane of the larynx and trachea, ultimately destroying the action of the lungs, by rendering them impervious. Acting from these premises, Dr. Wilson asserts, that our great indication is to clear the stomach and alimentary canal, as early as possible, by emetics and mercurial cathartics.

"Indeed, I believe these medicines to be the most efficient means hitherto known of speedily and effectually accomplishing, on any occasion, this important object." P. 409.

This plan will often, he thinks, check the disease in its nascent movements, and before actual inflammation has commenced; but we believe that such nascent movements, or in other words, the *spasmodic* stage, is rarely witnessed by medical men; and consequently, it is against the inflammation and exudation from the surface of the mucous membrane that we have principally to guard. Hence, while we take every opportunity of acting on the alimentary canal by emetics and calomel, we must detract blood from the throat, and counter-irritate in the neighbourhood, with all possible expedition and energy.

We have now presented a full analysis of Dr. Wilson's work. Its greatest fault, or more correctly speaking, its misfortune, is the diffuseness of the style. In one fourth part of the space, he might have given the same matter in an infinitely stronger point of view. Indeed, *medical economy* in medical literature is too much neglected. We are fully convinced, that the man who endeavours to diffuse professional knowledge on the easiest terms will, in the end, be the greatest benefactor to medical society. The higher classes have not time to peruse, and the inferior orders have not money to purchase voluminous publications; and hence the stream of science flows in narrow and extremely confined channels. We could illustrate this fact by anecdotes which would be scarcely credited by the well-informed members of the profession, but which are strictly true. We were lately in conversation with one of the first rate medical practitioners in the greatest city of the world, who neither knew the name nor any part of the writings of one of the greatest ornaments of medical science at the present period! It would be equally mortifying and discouraging to the really zealous cultivators of medical science, if they knew the narrow limits in which their labours move. It is true, that medical works take a large *circuit*, in consequence of medical clubs and societies; but the *actual readers* are few indeed!

This, in some degree, is owing to the expansion of little matter into large space, and to the consequent expence of purchase; so that, generally speaking, medical writings are little known, excepting through the medium of Journals.

But to return. The doctrine of morbid sympathy has, in Dr. Wilson, received a strong support and confirmation. Our readers are aware, that the leading points of our author's doctrines and opinions have been delineated lately in another publication, and the coincidence of ideas is a strong proof of the solid foundation on which the doctrines stand. We hope that Dr. Wilson will, in a future edition, compress the matter into less space, and adopt a more precise and philosophical style. There are some inaccuracies of language in the present work; but still it is a very valuable publication, and does infinite honour to its author.

---

### II.

*On the Nature and Treatment of Tetanus and Hydrophobia, with some Observations on a Natural Classification of Diseases in general.* By Robert Reid, M.D. Licentiate of the King's and Queen's College of Physicians in Dublin, &c. One vol. 8vo. pp. 136, with a Plate of the Spinal Marrow, as seen in Tetanus. Dublin and London, 1817.

In the Transactions of the College of Physicians of Ireland, vol. i. Dr. Reid has published a part of the present work, viz. that on Tetanus; and of this portion we gave some short account in the Medico-Chirurgical Journal for April, 1818. We may here state generally, that Dr. Reid found, in two or three cases of Tetanus, inflammation and effusion on the spinal nervous mass. Hence, he has been led to conclude, that as the natural functions are little affected in Tetanus, as well as the muscles and organs supplied by the nerves of sense, the spinal chord must be the original and principal seat of the disease, and that the thoracic and abdominal viscera are only drawn into disordered action subsequently. The appearances observed by Dr. Reid correspond with those of Dr. Sanders of Edinburgh, and some others, who are now investigating the pathology of the spinal canal; they are therefore entitled to great attention. We shall, however, pass over Dr. Reid's very ingenious and sensible remarks on a classification of diseases, according to the nervous masses by which the organs are supplied, viz. the brain, the spinal marrow, and ganglions; as also his investigations of the causes and morbid appearances on dissection, in order to concentrate our attention on the treatment of this formidable complaint—Tetanus.

From the rapid course of Tetanus, in general, it is evident that little time should be wasted in random experiment. The modes of treatment hitherto proposed are only remarkable for want of success. From viewing the inflamed state of the spinal contents, blood-letting might seem the principal remedy. But we know that by blood-letting the sanguiferous system in the brain is principally affected, and that the energy of this organ may be even so far reduced as to cause syncope. Dr. Reid believes that the cerebral system resists the disease of the spinal system.

"By abstracting blood, therefore, it is evident that we weaken one of our most powerful antagonists to the morbid action, and thus diminish our powers of resisting the general destruction." 65.

Experience has also, unfortunately, proved the inutility of general abstraction of blood, nor has this fluid exhibited the buffy coat so usually met with in all other inflammatory cases.

Traumatic Tetanus is commonly preceded by a cessation of inflammation in the wound, and hence many practitioners have endeavoured to re-establish the original inflammation by stimulant applications. But Dr. R. prefers the external inflammation of a blister to the surface of the back, as a more effectual remedy. This has been tried with success in a case related by Dr. Carter, in the Medical Transactions. There is another circumstance in favour of applying a blister along the whole length of the spine: we find that the disease, when chronic, has a natual tendency to throw out a serous fluid into the spinal canal, which is similar to the discharge of the blister. By exciting, therefore, such a discharge externally, it is evident we may prevent the internal determination.

"On the same principles, we observe also, that mercury may afford considerable relief in the chronic state of Tetanus, when employed in sufficient quantity, so as to occasion ptyalism."

So great a derangement has been often observed in the ganglionic viscera, during this disease, that many have been led to think that Tetanus arises entirely from the morbid state of some of the digestive organs. We must, therefore, perceive the necessity of employing purgative medicines freely, as has long ago been pointed out by Dr. Dickson. The following passage developes some of Dr. Reid's views; but we confess they do not appear very luminous to us.

"When we consider that muscular power appears considerably exhausted, after a long or violent exertion, and that most generally the effect of such exhaustion is profuse perspiration, we may conclude that, where profuse perspiration occurs in disease, the powers of the nervous system of the spine must be weakened. This we find to be the fact, as no disease is attended with greater debility than that accompanied with this symptom in excess. Now, as this disease [Tetanus] is the extreme of action in this system, which would become paralysis, if the effusion on the membranes enveloping the nervous mass were to amount to compression, it is evident our principal object in the treatment of Tetanus should be directed to weaken its energy. To procure free and copious perspiration would appear the most efficacious method of accomplishing this end.

"In the compound powder of ipecacuanha we have a preparation very powerful for this purpose; for, by this combination, the whole force of the opium, which is supposed so powerful in this disease, is directed to the proper point."

We either do not comprehend the scope of Dr. Reid's arguments in the foregoing passage, or we are unable to coincide with him. We believe that whatever good opiates, or opiates and sudorifics may have done in Tetanus, was by equalizing the balance of the circulation and excitement in the system, and thus relieving the part that was in a state of inflammation and super-irritation.

Dr. Reid considers that the cerebral system resists the encroachments of disease from the spinal system, and therefore ought to be supported in the due performance of its functions by stimulants, proportionate to the necessity of the case; "but not, however, to urge them, by any means, so far as to induce the contrary effect."

"Madeira wine is by far the best stimulant that can be employed, to support the energy of the cerebral system in Tetanus."

The quantity must, of course, depend on the effect; for it will often be wonderful how large a quantity of wine or spirits a patient can take in this disease, before it will have any perceptible effect upon him.

We now proceed to our author's observations on

### HYDROPHOBIA.

Cullen has accurately defined this disease in the following short sentence:—"*Potionis cujuslibet, utpote convulsionem pharingis dolentem cientes fastidium et horror.*"—We shall pass over the history and symptomatology of this dreadful disease, as, of late years, but too well known, in order to dwell more on the pathology. This last has hitherto, or at least till very lately, been involved in impenetrable darkness. Cullen observes—"I find that I can say nothing satisfying to myself, or that I can expect to prove so to others." Dr. Reid, however, thinks, that if the symptoms of this direful malady be investigated upon the principles which he has laid down relative to the different nervous systems which constitute the human frame, the phenomena which have been a long time observed in the disease can be clearly marked out, and their true nature distinctly understood. He observes, that it is evident, from the symptoms of the disease, that the ganglionic system is not engaged in morbid actions at the commencement. This system, therefore, is not primarily affected. The functions of the brain, too, he thinks, must be exempt from the *primary symptoms.* These last are diminished muscular action, shewn by the dullness, and disinclination of the patient to move. Every exertion is attended with tremor, which gradually increases until it amounts to irregular action. This is generally first observable in the muscles of deglutition. But as in the muscular structures no derangement can be detected after death, it follows, that the nervous system supplying these muscles must be the seat of the mischief. This is in the spinal marrow, Dr. Reid thinks. And here our author enters into a long, and not very fine spun hypothesis, which, as we cannot understand, so we shall not attempt to disprove. It may be correct; but certainly it must be incomprehensible to nine-tenths of the Doctor's readers.

In respect to the prevention of Hydrophobia, Dr. R. disclaims excision, as being "by no means even generally practicable." Now this we deny; and, on the contrary, affirm, that excision is very generally practicable—indeed, almost always practicable by a properly qualified surgeon. And we are convinced, that it is the only effectual preventive of the bite. Our author prefers the injection of boiling water into the wound, then filling it with Peruvian bark, and afterwards covering it with a poultice.

In the treatment, Dr. R. renounces venesection, though he acknowledges that inflammation obtains in various structures; but this inflammation is from diminished energy of the spinal marrow!

"Blood-letting has frequently been employed as a remedy: *but in no one instance is it asserted to have been attended with any advantage.*" P. 120.

Can Dr. Reid have forgotten the cases that were published some years ago, by one or two gentlemen in India, illustrating the good effects of venesection in Hydrophobia?

Our author recommends oxygen gas, as a remedy that supports the organic irritability of the heart; upon which the functions of the ganglionic system, in a great measure, depend. Our author also recommends the acetous acid on a curious hypothesis.

"When acetous acid has been taken into the stomach in small quantities, for a considerable time, the individual becomes emaciated, and a general paleness spreads over the surface of the body. The inhabitants of some inland counties in England have a curious mode of prize-fighting: two men agree for a wager to strike their uncovered heads with sticks, and the first that draws blood from the other is declared the winner. It is often wonderful what a number of blows they receive, which break the skin, and yet not one drop of blood escapes. The manner they accomplish this is by drinking quantities of vinegar, for several weeks previous to the engagement. This effect seems peculiar to the acetous acid, which appears to act thus by accumulating the blood in the venous apparatus.

"In Hydrophobia, the accumulation of blood in the venous apparatus is attended with considerable advantages. By means of this, the quantity and force of the blood thus impelled against the organs, which are weakened by the disease, is very much lessened: while at the same time, the powers of the sanguiferous apparatus to resist the disease, are preserved entire, not having been diminished by the operation of blood-letting. The acetous acid should therefore be given in repeated doses, at short intervals, until the patient is secure from the disease. Numerous cases of Hydrophobia have lately occurred on the continent, which are said to be cured by this remedy alone." P, 129.

If this remedy have nothing else to recommend it but the above-mentioned *ingenious* theory, we fear we are yet but little advanced in hydrophobic therapeutics. Upon the whole, we have been much disappointed in this volume of Dr. Reid's. It promised fair at the beginning; but, like most human undertakings, it fell off greatly as we advanced, and in the end, almost induced a fit of THEORIPHOBIA in our own persons.

Dr. Reid has talent for *pathological investigations*; and we would humbly offer our advice and opinion on this head to a man whom we respect. We are, ourselves, perhaps, a little attached to hypothesis; but we are resolved never to let the fairy region of speculation entice us, like the apples of Atalanta, from the safe and useful path of facts and observation.

Sat verbum sapienti.

---

### III.

*Observations, with Cases, illustrative of the Sedative and Febrifuge Powers of Emetic Tartar.*
By William Balfour, M. D. One Vol. Octavo, pp. 92. London, 1818.

Dr. Balfour justly reprobates the rage for hunting after *new* remedies, instead of improving the exhibition of *old* ones. We would rather say, that the English do not study *indications* with sufficient accuracy. We have plenty of medicines to answer all our purposes, if we only studied *when* and *how* to apply them with advantage.

"One thing must be admitted, for it is universally true, that the most skilful practitioners prescribe the fewest medicines. It is those who practise from mere authority, at random and by rote, who pour mixture upon mixture into their patient, and fly from medicine to medicine, in order to accomplish their purpose; thereby rendering the means of cure infinitely more insupportable to their patients than the disease itself." P. 4.

We fear there is some foundation, in fact, for the foregoing satire; but we were rather surprized at the following piece of intelligence from the intellectual city of Edinburgh.

"This much I can assert, that I have been in the habit, for many years, of meeting with the most eminent practitioners in this city, who never once hinted at the employment of antimonials, in even the most acute inflammatory diseases." P. 5.

It moreover appears, that Dr. B's professional acquaintances, merely acknowledge the excellence of emetic tartar, *in nauseating doses,* but that "the sedative and febrifuge powers of emetic tartar is a phrase quite new to them." Surely there must be a great "falling off" in the reputation of—emetic tartar, at least, since the days of

" our ain Cullen," for both he and his writings bear ample testimony to the sedative and febrifuge qualities of this far famed relaxer of spasm on the extreme vessels. They have only to look into the "Medical Primmer," Parr's Dictionary, and they will find both its sedative and febrifuge powers insisted upon in almost every page.

" In fevers of the *inflammatory* kind, and in *inflammations*, antimonials are alike the proper remedy." *Vide in verbo.*

We have given antimonials too extensive a trial in the phlegmasiæ, both by themselves and in conjunction with venesection, to be carried away by the opinion of Dr. Balfour, " that in many cases of local inflammation accompanied with violent re-action, blood-letting, to one-third the extent generally practised, is not necessary to the cure; that a *speedy and perfect* cure can be obtained with the loss of so moderate a quantity of blood, as to warrant the conclusion, that it might be safely omitted altogether, even in circumstances in which it is generally considered the only means of saving the patient." P. 8.

We have heard so *much* and experienced so *little* of these *speedy and perfect* cures by particular remedies and processes, that their mention produces something like the sedative effect of an antimonial, in a *nauseating dose*, upon our intellectual stomachs. We agree with our author, however, in the following sentiments.

" It must be recollected I am not combating blood-letting *in toto;* I only object to its being carried beyond just bounds; to its being employed *singly and alone*, (a piece of tautology by the bye) in any important case."

We refer to page 106 of our present volume for similar sentiments.

Nine-tenths of Dr. Balfour's volume is filled with cases, illustrating the sedative and febrifuge effects of emetic tartar in pneumonia, inflammatory gout; in rheumatism, chronic and acute; in cynanche tonsillaris; in idiopathic fever; in hernia humoralis; in chronic inflammation of the bladder, &c.

We are not, upon the whole, so much disposed to question the justness of Dr. Balfour's inferences, as the originality of his proposals. We are confident that every medical practitioner on this side the Tweed, of any pretensions to observation or experience, is well acquainted with the sedative and febrifuge qualities of emetic tartar. No man who has ever taken an emetic himself, or seen another under that *sedative* operation, but must be of this opinion. We believe, indeed, with Dr. B. and we have elsewhere expressed that belief, that antimonials have been too much neglected in this country, since purgation has come so much into vogue. But this is the case with every new improvement. It absorbs for a time all our attention, and things of value are, in this interval, apt to be overlooked.

---

### IV.

*A succinct Account of the Contagious Fever of this Country, exemplified in the Epidemic now prevailing in London: with the appropriate Method of Treatment, as practised in the House of Recovery, &c.* By Thomas Bateman, M. D. &c. &c.

A public Institution, such as that over which Dr. Bateman presided during a long period of fourteen years, is certainly well calculated to afford materials on which to ground the most important conclusions. Whether our author turned his talents and his opportunities to the best possible account, it is not for us to say. We have no right to examine the mouth of a "gift horse;" and what the medical officers of hospitals are pleased to communicate, we must be contented to receive. It may possibly be true too, as our author observes, that

"If we put aside all hypothetical speculations, and controversial discussions, and confine our attention to points of real practical importance; to the symptoms and their changes; to the operation of the remedies which we employ; and to the appearances of organic derangement left by the disease, the subject is reducible within very narrow limits." *Pref.* ix.

We agree indeed with Dr. Bateman, that fever is not that complicated disease which it is often represented to be, and that its treatment admits of considerable simplification, "the indications being generally less obscure than in many other diseases, and the operation of remedies clear, and often effective." *ib.*

The almost constant concurrence of dearth and pestilence is proved by the history of all nations. The present epidemic, like its innumerable predecessors, has arisen in or after a season of scarcity among that portion of society who, from manners and habits, are peculiarly obnoxious to the ravages of fever.

Dr. Bateman is of opinion, that deficiency of nutriment must be the principal source of epidemic fever, since the closeness of the habitations of the poor, the uncleanliness of their persons, furniture, and apparel, and the accumulating filth in the lanes and alleys which they occupy, remain unchanged in all seasons; whereas epidemic fever appears but rarely, and with long intervals of absence. We are very far, however, from acceding entirely to these conclusions of our author, since we know that in the West Indies, for instance, epidemics rise and disappear, totally independent of any thing like deficiency of nutriment, but from certain constitutions of the air which we cannot yet ascertain.

Here Dr. Bateman takes occasion to oppose the doctrine of Dr. Bancroft, respecting the non-possibility of a contagious or epidemic fever being generated by the morbid and even natural effluvia of the living body, when allowed to accumulate by want of cleanliness and air. And surely his admission of this possibility, militates against, if it do not entirely overturn his former position, relative to the uncleanliness of the persons of the poor, &c. never producing fever.

The present epidemic took its rise at a period of unparalleled distress among the labouring poor,

"When the loss of employment, occasioned by the termination of the war and the general suspension of the manufactories, concurred with the failing harvest of 1816 to increase the difficulties of procuring subsistence."

In this place Dr. Bateman takes an opportunity of adverting to the strange and monstrous doctrines of Dr. Maclean, to which we directed the attention of our readers in the last number of this Journal. We wonder, however, that Dr. Bateman should have deemed it necessary to take any *serious* measures to refute the crude speculations of Dr. Maclean.

It appears, from our author's experience, that at all times there is a greater disposition to fever in London during the autumnal months, which diminishes with the approach of winter, for few patients have, in general, claimed admission into the institution in the months of January and February.

In respect to the *denomination* of fever, our author's experience has tended more and more to impress him with the conviction of the identity of that disease under all its modifications. To this conclusion our own experience has led us many years ago. It is true, as Dr.

Bateman observes, that the character of fever varies with the different circumstances in which it occurs; with the age, constitution, and previous health of the patient, and with the intensity of the exciting causes. Still it is not more varied than other febrile diseases, as the small-pox, scarlet fever, &c. under similar circumstances. Examples of the most distinct modifications which it undergoes, are often observed in individuals of the same family.

"Thus," says Dr. Bateman, "in the instance of a man and his wife, who were brought to the House of Recovery together; the former was affected with the mildest symptoms of fever, which scarcely confined him to bed, and terminated in speedy convalescence; while his wife was lying in a state of stupor, her skin covered with *petechiæ* and *vibices;* in a word, exhibiting the most formidable symptoms of the worst form of typhus. Yet these extreme degrees of the disease manifestly originated from the same cause; and it would be equally unphilosophical to account them different kinds of fever, and give them distinct generic appellations, as in the case of the benign and confluent small-pox, which are generated in like manner, from one contagion." P. 23.

This doctrine, it is well known, is maintained by some of our best writers on fever; especially Dr. Armstrong, whom Dr. Bateman handsomely allows to be "the author of one of the best treatises on typhus that has ever appeared." The able physicians, too, who are attached to the various fever institutions in the sister island, and who have had ample opportunities of investigating the nature of fever, seem to have come to similar conclusions. The following passage strongly corroborates the opinion, that the impression of the febrile causes is first made on the *nervous* system, and that the *vascular* derangement is a secondary event, in order of time.

"On the most cursory view of the patients occupying the wards of the House of Recovery, two very remarkable characteristics of the disease impress themselves upon the attention; namely, the universal expression of debility exhibited in the countenance and supine position of the patients, and the general absence of preternatural heat. On a closer inspection, the small, weak, and hurried, or irregular pulse, and the disturbance of the sensorial functions, still further develope the character of the disease. Though but few instances of considerable delirium or great confusion may present themselves; yet the dullness, deafness, depraved sensations, complaints of disturbance of mind, frightful dreams, and innumerable distressing feelings and noises in the head, manifest the morbid condition of the sensorium. P. 28.

Dr. Bateman thinks, that the classifications of Armstrong and Percival come, upon the whole, nearer to nature and truth than those of any preceding writers.

To the *third* subdivision of typhus proposed by Dr. Armstrong, our author objects; viz. the *congestive typhus,* as founded on hypothetical principles. On this point we disagree, *in toto,* with Dr. Bateman. We have seen numerous cases of the congestive typhus totally different from the state of collapse supervening on protracted fever, and where, as Dr. Armstrong has observed, the congestion of the viscera could not be overcome by a proper degree of excitement. Here we must lessen the quantum of blood in the system, *coute qui coute,* and endeavour to bring on a salutary reaction. However formidable this reaction may be, it is much more manageable than the prostration and oppression of the congestive stage. Dr. Bateman saw only two cases which approximated to this character, as drawn by Dr. Armstrong, and in both these cases, "this condition supervened at that latter period of the disease in which the more formidable symptoms of fever usually arise, and apparently during a stage of excitement." P. 32. From this quotation, it is clear that Dr. Bateman either never saw a case of congestive fever, as drawn by Dr. Armstrong, or that he never noted it. We appeal to the observation of the profession on this point, and we protest against the justice of the epithet "hypothetical," as applied by Dr. Bateman, to a state or condition of fever which actually exists, and which, in fact, has been observed not in typhus fever alone, but in the graver types of fever in every quarter of the globe. We refer to the writings of Dr. Jackson on this subject; and we may instance the description of a peculiar species of congestive fever recorded by Dr. Archibald Robertson, as noticed in the July Number of this Journal, page 73, et seq.

Dr. Bateman, therefore, adopts the plan of two subdivisions, the simple and the complicated typhus; and under these two heads proceeds to delineate the character of the epidemic.

### I. Simple Typhus.

The mildest and least complicated form of this division occurred in children, commencing with lassitude, chills, and head-ache, succeeded by heat, dry skin, thirst, aching pains in the loins and limbs, oppression at the præcordia. Even when neglected, these symptoms frequently subsided spontaneously after a week or ten days; and they invariably did so at the House of Recovery by the assistance of the cool atmosphere, and the treatment of that establishment. This form was almost exclusively confined to children, or persons under seventeen years of age; while the parents or adult members of the same family were afflicted with the most formidable symptoms of typhus, or even had died by its attack. It was only in a very few instances, accompanied by that pungency of heat on the surface which would indicate the propriety of the cold affusion.

A considerable number of adult patients experienced this simple form of fever, though not so mild as that just alluded to. The general disturbance of the functions was greater, the pains more acute, and the march of the disease more protracted. In most cases the attack was marked by an immediate depression of strength, with chilliness often amounting to shivering, a sense of load and oppression about the præcordia, and a total loss of appetite.

"These symptoms have been speedily followed, or actually accompanied by head-ache, most commonly referred to the forehead, or occiput, and by severe aching pains in the loins and limbs, especially in the larger joints, which were much aggravated by every attempt to move. The skin now became hot and dry, without any disposition to perspiration; the pulse rising in frequency, commonly to 100 or 110, but varying from 86 or 90 beats to 130 in the minute. The tongue, in many cases, was covered with a moderately thick grey fur, rather clammy; in others it became extremely dry and parched, or glazed, and sometimes slightly tumid, but with very little appearance of fur, and of a dusky red, or yellowish hue, and sometimes brown. The thirst was generally considerable, and much complaint was made of the constant bad taste in the mouth, and the parched and heated condition of the fauces. The nights were generally passed without sleep, or with some of those disturbed and dreaming slumbers, in which the activity of the imagination so nearly equals the waking reverie, and there is so little actual repose, or feeling of refreshment on waking, that the individual is unconscious of having slept; but in general, the patients were free from delirium, even during the night. The bowels have commonly been disposed to constipation in the beginning, but have generally continued free afterwards, with little more assistance than the common saline mixture afforded." Page 39.

The above is a fair specimen of our author's symptomatology, and conveys his portraiture of simple typhus. It will readily be granted, that there is nothing in it which evinces originality of thought, or superiority of description. It is an inanimate picture, and affords little proof of being conceived—" in the lusty stealth of *Nature*."

In Dr. Bateman's subsequent remarks upon particular symptoms and phenomena he is more happy, and evinces, upon many points, considerable acuteness of reasoning, and accuracy of observation. In his remarks on the state of the tongue in fever, we do not entirely coincide, as we think he has not connected its different appearances sufficiently with the phenomena in the digestive organs of which they are indications. We confess, however, with Dr. B. that the tongue, as well as the pulse in fever, is still *a res fallacissima,* and, therefore, the state of this organ should always be taken in conjunction with various other concomitant phenomena in the system at large.

The only variations in the functions of the alimentary canal, which Dr. B. has been enabled to observe were, some nausea and vomiting, especially on taking food; sometimes a disposition to diarrhœa, the bowels also being excited to immediate evacuation by food or drink. These symptoms, however, commonly occurred in the second week or later, and appeared, we think with reason, to our author, as the consequence of an increased irritability of the alimentary canal, from a morbid condition of its secretions.

"Under all the modifications of fever, but more in this simple form, the skin remains dry, and free from any marked tendency to perspiration." P. 47.

During the hot weather of last summer, we observed a very considerable number of fever patients in the House of Recovery, with perspiration on their skins, during the course of the fever, but without bringing any alleviation of the symptoms. We observed, however, that although there was *perspiration,* there was *not* a perspirable state of the skin and cutaneous vessels. The latter had always a harsh, unnatural feel, though covered with a fluid which appeared rather to ooze out than come forward as a secretion. This distinction is a point too little attended to.

"A spontaneous perspiration, however, not unfrequently occurs, and, in a few instances, affords perhaps the only example of a marked natural crisis that is ever witnessed at present." P. 48.

Here too we cannot fully agree with Dr. Bateman. Our opportunities of seeing fever in all its forms have been not few: but we could generally observe a revolution, as it were, in various functions, and especially in the state of the circulation and sensibility, as the fever took a turn. It is true that a *sudden crisis,* marked by diarrhœa or other evacuation is comparatively rare, yet the discriminating eye will perceive the change, and generally find it marked too, by some evacuation from the system, either secretory, excretory, or exhalent. Deafness was observed by our author to be, upon the whole, a favourable symptom; we have found it so, and have sometimes thought that it was a *cause* as well as *symptom* of safety. It conduced to keep quiet or unexcited by *external* impressions the great sensorium; and this probably is a matter of no small importance in a disease which, by some of our ablest physicians, has been looked upon as a species of cerebritis.

### II. Complicated Typhus.

Under the preceding mild form of fever, two-thirds of the cases proceeded safely to convalescence; but there was a considerable tendency to relapse; for out of 678 cases, 54 had returns of typhus. Our author does not attribute these relapses to the re-operation of contagion; and in this again we differ from Dr. Bateman. We have reason to believe, that since greater attention has been paid to ventilation, and other means of dissipating contagious miasmata in the Institution, there has been less tendency to relapse.

"The character most frequently exhibited by the fever, under its more violent forms, which I am about to describe, approximates very closely to that of the 'slow nervous fever,' so accurately depicted by Dr. Huxham; a fever which manifestly differs from the 'putrid pestilential fever' described by the same able author, only in the less violence of its symptoms, and its more protracted course." P. 52.

In the latter part of this quotation we fully coincide with Dr. Bateman, and sincerely hope that future authors will follow his example in divesting fever of so many different names and species, convinced, as we are, that the different *forms* depend entirely on the difference of remote or exciting causes; the idiosyncrasies of constitution, and the organs on which the violence of the febrile movements falls, from previous predisposition to disease.

In complicated typhus there was, in general, no very great difference from simple typhus till the eighth, ninth, or tenth day, or even later, when a considerable increase of languor and debility was felt and complained of, and was very obvious to the attendants.

"The voice became more feeble, and the speech slower, the tongue dry, with a brown streak in its centre; the pulse smaller and more compressible, (but quicker and with a slight sharpness in its beat); the eyes dull, and the countenance heavy, and the patient sunk more decidedly in the supine position. This change was speedily followed, or actually accompanied, by a more manifest disturbance of the sensorium, and of the functions dependent on it. The first sign of this disturbance was a confusion or wandering of the mind, especially in waking from slumber; this at length increased so as to continue through the night, or even during the day. In many cases this intellectual disturbance was an approximation to stupor, rather than to excite delirium; and dullness or inaptitude to receive external impressions. With this state of confusion and slighter stupor, there was often a considerable degree of drowsiness, particularly in the day time; sometimes a great disposition to moaning; though the patient was commonly unable to refer to the particular seat or cause of complaint. The skin continued parched, but not hot; and the tongue brown in the middle, and dry along the edges; one cheek exhibiting a circumscribed flush, and the pulse beating about 120 times in the minute, generally small and easily compressed, but with a sensible smartness or slight jerk in its stroke.

"In this state patients remained two or three weeks, where active remedies had not been resorted to, without any material increase of the symptoms, and have been slowly, and with difficulty restored where proper measures were adopted, or sunk from mere exhaustion of the vital powers, without any supervention of more severe or malignant symptoms, without subsultus or stupor." Page 55.

In adverting to one or two cases which seemed to assimilate with the congestive fever of Armstrong, our author candidly acknowledges that he is "much disposed to believe that these symptoms are justly referable to a state of venous congestion, with feeble or oppressed action of the heart and arteries;" but, probably with justice, attributes them to "one of the various irregularities or disturbances in the balance of the circulation, which constantly occur in the progress of febrile excitement." He adds judiciously, that "it is more easy to mark these, and more important to ascertain the appropriate modifications of treatment which they require, than to explain them upon any satisfactory principle."

Our author observes, that these irregularities in the balance of the circulation, which occur in the progress of fever, contribute materially to modify its forms as well as treatment. They sometimes take place on the second or third day, more frequently, he thinks, in the second week, or still later towards the close of the fever, when some of the important organs became unusually disturbed in their functions, manifesting signs of greater determination of blood, or of a certain degree of inflammatory action. The brain and the respiratory apparatus appear to be most liable to this partial injury; "but not unfrequently the intestinal canal, the liver, the peritonæum, the tonsils, the trachea, and even the bladder, suffer in a similar manner." P. 61.—From this passage it is obvious, and indeed Dr. B. subsequently acknowledges it, that he does not subscribe to the theory of the learned and ingenious Dr. Clutterbuck, relative to the seat of fever. Where the functions of the sensorium were much disturbed, Dr. Bateman conceives, that some degree of inflammatory or sub-inflammatory action must have been going on in that organ; and when jactitation of the limbs, and rolling of the head, were superadded to the usual phenomena of delirium, he thought there was little doubt of "*acute cerebral irritation.*" This form of the disease was more common among the better classes of society than among those who constitute the general mass of hospital practice.

Complication of pulmonic affection and fever occurred very often in the present epidemic, and especially during the winter months. No less than 144 out of 678. These inflammatory affections of the respiratory organs, occuring in the graver forms of typhus, were most unmanageable and even fatal, when the trachea and larynx became their seat.

Among the formidable and fatal of the accessory diseases, which occasionally supervened in the latter stages of fever was erysipelas, affecting the face and scalp. Three out of five cases, which occurred to Dr. Bateman, terminated fatally.

The period of convalescence commonly bore some proportion to the duration of the disease. The tongue generally remained white, and the pulse rather quick, for some days after the patients were able to move about; and in this period, very slight errors in diet, exertion, or exposure to cold, induced relapse.

However different this fever may appear from the putrid pestilential fever of Sydenham and Huxham, yet, as it is still the result of the same circumstances, and recurs with every return of public distress, so Dr. B. considers it as doubtless the same disease, "stripped of its malignity by all those concurring changes in the arts of domestic life, and by those public provisions for promoting the general health, which are among the most important improvements of modern polity."

In respect to the post mortem appearances, Dr. Bateman's experience is extremely limited; since, during the present epidemic, "only one investigation of that sort has been instituted, at the special request of a medical friend." P. 86.

### Treatment.

The humoral and Brunonian hypotheses, as Dr. Bateman justly observes, have, in disparagement of the Hippocratic maxim — "*contraria contrariis medentur,*" had

"The effect of fixing an impression, which, at this day, is but too manifest, not only in the popular feelings and prejudices, but in the minds of the mass of medical practitioners, unfavourable to the cool and antiphlogistic management of this disease. That this impression exists in considerable force, in a multitude of practitioners, is evident from the resort which is so frequently witnessed, *even in the early stage of fever*, to the camphor mixture, cordial confection, and ether, and the subsequent use of bark and wine; from the disinclination to blood-letting and free purgation, and to the external and internal use of cold liquids." P. 90.

This is a melancholy picture of metropolitan practice, after all the light which has been thrown on the pathology and treatment of fever by the laborious experience of our army and navy practitioners, as well as by a host of able men in our provincial towns and cities! Can this be wondered at, however, when we read the following confession from a man of such ability and philosophy as Dr. Bateman?

"I believe there are few physicians who, like myself, commenced their professional career, impressed with the doctrines that prevailed in the schools at the close of the past century, in which the terror of debility was certainly predominant, who will not acknowledge that their subsequent practice *has been a continued struggle between the prejudices of education and the staring conviction of opposing facts, which were continually forcing themselves upon their observation;* and that they have more especially been compelled to a gradual but material change in their views respecting the use of the lancet, not only in fever, but in other diseases. I am fully conscious of the extent to which my own practice has been cramped by this prejudice, and of the reluctance with which I have admitted the evidence of my senses, till frequent repetitions, and the sanction of other authorities, had rendered it irresistible" P. 98.

This is a humiliating portrait of medical philosophy, and may abate at once, and most effectually too, the pride of science—at least of *our* science! It shews the *extent* to which prejudice will go, and it clearly evinces the necessity of perseverance in the work of demolition, till not a stone in the temple of scholastic error shall tell where its doctrines *lie.*

"Gutta cavat lapidem non vi, sed sæpe cadendo."

Dr. Bateman judiciously observes, that whatever difference of opinion may exist relative to the local or general inflammatory excitement at the commencement of fever, yet that our great object is to prevent the occurrence or increase of this in the course of the disease; "and the only practicable means of anticipating this event, or of speedily abridging the term of the disease, are such as enable us to subdue other inflammatory disorders." 92.

Under all circumstances, in hospital practice, the prompt evacuation of the stomach and bowels should be the first expedient. A scruple of ipecacuan, combined with a grain of tartrite of antimony, will act upwards and downwards; but our author thinks it better to exhibit the ipecacuan alone, and follow it up with five grains of calomel, and six or eight of jalap. In the following sentiments we fully coincide with our author.

"An emetic, whether by the direct sympathy between the skin and the stomach in a state of nausea, or by the general stimulus given to the arterial system by the concussion of vomiting, seems to possess some power of equalizing the circulation and determining it to the surface; thus, at once relieving the external chill and the internal oppression. This effect may be augmented by the use of the pediluvium, or where it is at hand, the tepid bath, and by the administration of warm diluent drinks." P. 94.

When the stage of excitement has come on, more active antiphlogistic measures may still cut short the fever, if they are resorted to on the third, fourth, fifth, or even sixth day; these measures are venesection and the cold affusion. Dr. B. does not entertain a high opinion of the cold affusion; and now, with most other metropolitan physicians, has substituted the sponge for the bucket or shower-bath.

—"After having found by repeated trials, that the *latter*, with all the disadvantages of the inconvenience, fatigue, exposure, and alarm connected with them, produced, in fact, little more than the temporary alleviation of certain symptoms, which followed the use of the sponge, at least after the third or fourth day."—"I lately (says our author) witnessed the complete and immediate extinction of the fever in two cases, not in the House of Recovery, by a single blood-letting, the one performed on the fourth, the other on the fifth day." P. 99.

Dr. Bateman, with much accuracy of discrimination, observes, that if, on the third or fourth day, the headache be acute; or if, without severe head-ache, there be much watchfulness, a hurry of thought and rapidity of speech, and an unusual sensibility to light, especially if the pulse be 110 or 120; such symptoms mark a condition of the sensorium bordering on inflammatory action; and blood-letting is the most prompt and effectual mode of anticipating the morbid changes which are likely to follow, and which sometimes come on so suddenly, as to inflict an irremediable injury on that delicate organ the brain. This often takes place by the ninth or tenth day, and frequently gives little alarm till unconquerable evil is done. Local blood-letting affords but partial and transitory relief. A well-opened vein is the proper exit for the blood, and effectually checks the rising inflammation or congestion of an organ.

"No appearance of languor and debility should induce a disposition to swerve from a steady pursuit of the antiphlogistic plan, in diet, regimen, and medicine. The frame and physical energies of the patient cannot have been, as yet, impaired, or even partially exhausted; they have merely sustained a sudden depression, from which they speedily recoil, if the oppressive load of the disease be quickly removed."

This is well expressed, and conveys an important truth. We need not say how often we have urged this doctrine in the pages of this Journal and elsewhere. Our author directs the bowels to be kept open daily, or every second day, by means of calomel, with a little rhubarb or jalap, or the sulphate of magnesia.

In the simpler forms of fever, rhubarb alone is a useful, mild, and efficient laxative; but, in all cases, it is well to clear the alvine canal effectually, by the occasional addition of three, four, or five grains of calomel.

"The saline mixture, containing a couple of drachms of the acetate of ammonia, operates itself, in some cases, as a mild aperient; and that, or some other neutral saline, should constitute the principal medicinal treatment of fever, if not through its course, at least at the early and middle periods, unless some particular pressing symptom should require attention." P. 103.

Whatever the apparent degree of debility at this time, our author judiciously, we are sure, reprobates the exhibition of camphor, etherial fluids, aromatic confection, and every description of cordial and tonic, and especially the cinchona. Cool air, and a corresponding antiphlogistic regimen are, of course, necessary on these occasions. In the following sentence, we fully coincide with our able author.

"The introduction of camphor, aromatic vinegar, or other fragrant substances, or odoriferous fumigations into the patient's room, is certainly to be deprecated. These matters have not the smallest influence in destroying the contagious or offensive effluvia; they only conceal them, and render us less sensible to their presence: whereas, *under a free ventilation*, all offensive exhalations are dissipated, and that most grateful condition of the atmosphere of a sick room is produced, in which no odour whatever is perceptible on entering it." P. 107.

We would apply precisely the same reasoning to acid fumigations. If free ventilation can dissipate contagion, what more do we want? We repeat here, what we have many times said, that we have no proof that acids can destroy contagion; whereas, we have the most unerring evidence, that atmospheric air can render it completely inert. We are surprised to hear so sensible a man as Dr. Bateman, talk of using fumigations, where ventilation cannot be effected. Where is it that ventilation—*free* ventilation, cannot be put in force? If the hold of an hundred gun ship can be perfectly ventilated, what habitation in the British empire can have an excuse for a stagnant atmosphere. If such places there be, they ought not to be permitted to exist; but we have seen no such places. We have repeatedly perambulated every lane and street in St. Giles's, and occasionally descended into the subterranean abysses of poverty, vice, and sickness, but we have not met with any locality where the three elements of water, fire, and air might not be made to permeate every sinuosity of filth, and root out every particle of contagion. On this point of Fever-Hygiene, then, we cannot agree with our author; and much do we suspect that there is yet a nook or cranny in Dr. Bateman's mind, from which the pure atmosphere of his own senses and experience has not been able to eradicate the contagion of preconceived and authoritative doctrines. The light, however, which is spreading over the medical horizon, will, ere long, dissipate this and other nebulous specks from the mirror of Nature; the only surface that can fairly reflect the true features of medical science. What Dr. B. has said of *antimony* might probably apply to fumigation.

"My knowledge of the remedial powers of antimony [fumigation] is rather a matter of tradition, the gratuitous admission of a *received opinion*, than the conviction of experience."

Now, with all due deference to Dr. Bateman's experience, we do humbly beg leave to state from *experience*, and not from received opinion, that antimony is a powerful remedy in fever, especially when a rush of blood is threatening to overwhelm some interior organ. The nausea resulting from this remedy effectually lowers the action of the heart, and checks the tide of blood, for a time at least; and in fever, complicated with pneumonic inflammation particularly, we should feel much embarrassed, had we not antimony to aid the lancet. Opiates we condemn in fever as much as Dr. Bateman, when employed alone; but in combination with calomel and antimonial powder, it forms an important item, especially when any organ or tissue is verging towards inflammation or congestion.

It is in these complications, as Dr. Bateman has properly observed, that the method of treatment becomes more important, and more difficult of decision. The condition of the sensorium, the vascular, nervous, muscular, and organic systems, must be particularly examined, as well as the age and constitution of the patient, in order to guide us in the application of appropriate remedies. In active delirium, with rapid and continued talking, moaning, or occasional cries of distress, "some evacuation of blood is absolutely necessary, whatever the state of the pulse may be." "The temporal artery may be opened with great advantage under these circumstances." Dr. B. considers, and we think with reason, that venesection is as good as arteriotomy; and, of course, it is much more practicable. Dr. B. speaks very slightingly of leeches to the temples, and in this our experience has been very different from his; for, in reality, we trust more to this species of evacuation than to general blood-letting in phrenitis, of which there is always more or less, under the circumstances pointed out above. We agree with our author, however, in the great utility of cooling evaporating lotions to the shaven head, as a mean that very effectually controls the local turgescence of blood, and the inflammatory reaction which thence results. Blisters to the back of the neck are important remedies, after evacuations from the vascular system, and during the cooling process going on over the surface of the scalp.

Even in the later stages of fever, when inflammations and congestions have established themselves—when "the supine and helpless condition of the patient; his constant and feeble mutterings when spoken to; his dull and sunken eye; his dark and trembling tongue; his starting tendons; and his involuntary evacuations, have all been deemed indicative of a great general prostration of the vital powers, and consequently of the necessity of active stimulation, as the sole means of counteracting the tendency to death;" still we must avoid the Brunonian practice, and deplete in some shape or other—especially by local detractions of blood, blisters, and attention to the bowels. Dr. Bateman has found, that even the smallest quantity of undiluted stimulus was injurious—"*whenever the tongue remained parched, the skin dry, and the pulse above* 120, *with the slightest perceptible sharpness in its beat.*" 121.

In cases of that sudden collapse which occurs most commonly in patients past the meridian of life, and the symptoms of which are sufficently unequivocal to be distinguished by an attentive observer, Dr. B. recommends a liberal though cautious administration of wine. In these instances, the increase of languor and actual debility is very manifest; the sense of faintness and sinking, with oppressive respiration, being very distressing. The patient generally lies supine and motionless; the skin becomes cool, and for the most part damp, having a relaxed, chill, and clammy feel; the tongue, though previously brown and dry, becomes moist, yet remains loaded; and the pulse usually increases in frequency, but is so feeble as to feel rather like an undulation than a stroke. Here the patient will inevitably sink, if stimulants be not duly supplied.

Dr. B. thinks, that in certain cases of typhus, where the disease is protracted by a state of diarrhœa, with little affection of the sensorium, but with a small and feeble pulse, brown, but not very dry tongue, and great languor and debility, wine may be advantageously given, when the cretaceous mixtures, with laudanum, fail to remove the complaint. In respect to bark, we were greatly rejoiced to hear a London Physician, of Dr. Bateman's known ability and candour, utter the following sentiment:

> "It is to be wished, however, that this substance were erased for ever, from the catalogue of medicines employed for the cure of this disease." P. 129.

In typhus, complicated with pulmonic inflammation or congestion, Dr. Bateman was disappointed in the use of the lancet. This disappointment arose, we are quite convinced, from not carrying the measure to a full extent. A few years ago, we had peculiar opportunities of giving venesection the most decisive trial in pneumonia typhoides occurring in debilitated and depressed prisoners of war, in this country; and we are satisfied, from repeated evidence, that early and decisive evacuations from the vascular system, are the only effectual means of subduing the disease.

We shall not enter on the subject of contagion, since there is nothing in the work before us which Dr. Bateman has not already published through the medium of Rees's Cyclopœdia.

Our author never once alludes to the exhibition of calomel in typhus, with the view of producing constitutional effects, and thus restoring the secretions, and the lost balance of the circulation. This is a great defect in the work, and an indubitable proof of the "extent to which his own practice has been cramped by prejudice, and of the reluctance with which he has admitted the evidence of his senses, &c." We acknowledge, however, that in this metropolis, the purgative are more beneficial than the constitutional effects of calomel.

Upon the whole, we are more disposed to *approve* than to *applaud* the work before us; it is more indicative of judgment than of genius—of cautious prudence than of fortunate boldness. It is highly entitled to *negative* praise, being nearly free from fault, though not superabounding in merit.

---

### V.

*An Inquiry into the Influence of Situation on Pulmonary Consumption, and on the Duration of Life; illustrated by Statistical Reports.* By JOHN G. MANSFORD, Member of the Royal College of Surgeons of London. 8vo. pp. 135. London, 1818.

THIS book is an attempt to prove the injurious influence of all elevated and maritime situations on pulmonary consumption, and appears to be put forth with the professed object of persuading all our phthisical patients to seek for the cure, or mitigation of their malady, in the dense atmosphere of inland vales—and especially in the vales of Somersetshire. The South of France, Italy, Sicily, Malta, Spain, Portugal, Madeira, Devonshire, and Cornwall—in short, all the places more commonly re sorted to by the unhappy victims of pulmonary disease, Mr. Mansford delineates as highly deleterious, either from their height above, or their proximity to the sea, or some other quality or qualities to which the happy vallies of his own neighbourhood are fortunately strangers.

"Every place (he observes) which I have had an opportunity of examining, confirms me in the opinion, that situations, uniting all the desired local advantages, are very few; and in proportion to the difficulty of finding such, should be the value attached to that, whose comparative exemption from consumptive diseases is established upon the most unequivocal evidence. A salutary retreat may here be found at all seasons of the year, and especially during the winter and spring months, from the mildness of the air, and the shelter afforded by the Mendip hills; which, like a stupendous wall toward the north and north-east, afford an effectual screen to the winds blowing from those points, which, especially in the spring, are generally the most prevalent." P. 53-54.

"To these advantages of a physical nature, may be added others of a more obvious and inviting character. The varied and romantic scenery of the neighbourhood cannot fail to charm those who possess a relish for the beauties of Nature; while the tastes and habits of individuals may be gratified in the society of a city, or the seclusion of a village." P. 50.

"How many parents are there (he exclaims in another place) at this moment suffering under anguish of recent, perhaps of repeated losses from this devouring disease; trembling for the fate of the one which appears marked out for the next in succession, at whose vitals the monster's dart is already pointed, and eagerly looking round for directions what step to pursue, uncertain whether to remain or fly—and if to fly, whither to go! To such I would exclaim, with all the earnestness which a conviction of its importance must naturally excite—fly—fly, if it be in your power, and before it is too late, from the fatal spot. It may be death to remain; there may be safety in flight. Fly then to some more favoured situation, answering in its character to those which have been described. If you know not where else to find such a one, (and there are probably not many) go thither" [to the vales of Somerset, to wit;]—"Go thither without delay." P. 41.

Now we are afraid that our readers may consider all this as showing something too much of "*the influence of local attachment*;" so well sung by one of the west-country bards. And it must be admitted, that, when we take these and other similar passages of this essay, in conjunction with the author's declaration in the Preface, that he writes for the public at large as well as the profession—and call to mind an unhappy conspicuous feature in the literary character of the present age—there appears some excuse, at least, for entertaining some misgivings of this sort. We trust, however, that such feelings are gratuitous and unfounded; and we advert to them merely to point out to Mr. Mansford (whom we know to be a gentleman of good acquirements) the caution necessary to segregate the respectable part of the profession from the swarms of advertising quacks that daily inundate the medical world with puffs of every quality, form, and size.*

From this seemingly ungracious exordium, neither our author nor our readers must imagine that we are going to dismiss the production before us, without more mature and particular notice. The importance of the subject treated of is alone sufficient guarantee against so cavalier a mode of proceeding. Than this very subject, indeed, we are of opinion, that the whole compass of medical literature does not afford a single one of more interest and importance. The consideration of the innumerable and omnigenous medicines and remedial measures that have been employed in consumption—and the almost equally unsatisfactory results of all—compel us to come to the painful conclusion, that, if this disease is not, as Bayle maintains, absolutely incurable, it is, at least, so little so, as, in our estimation, to give to measures that hold out any prospect of prevention, an interest and importance which no new remedy or mode of treatment can ever, in these later times, excite in the breast of the experienced physician. In this, above all other diseases, we find every successive generation, we might almost say every successive author, holding up to disregard, if not to ridicule, all anterior modes of treatment; and if it happens—as is too often the case—that something new is offered to our notice, which the partial proposer fondly imagines to be founded on the stable basis of a more chastened experience and more enlightened pathology, the history of a very few years in recording the fortunes of the vaunted novelty, only reiterates the inglorious doom that awaited its precursors !

> Sic volvenda aetas commutat tempora rerum,
> Quod fuit in pretio, fit nullo denique honore;
> Porro aliud succedit, et e contemtibus exit,
> Inque dies magis appetitur, floretque repertum
> Laudibus, et miro 'st mortaleis inter honore.

In making these observations, we beg not to be misunderstood as undervaluing the utility of medical treatment in consumption ; much less as seeking to weaken the

---

* The Editor begs that the author of the work under consideration may bear in mind that *he* is not the writer of every critique in the Journal, and consequently, that he is not to be identified with every censure therein contained. He never, however, admits anonymous criticism ; and never confides a work for review to any but men with whose probity and talents he is himself acquainted.

faith of our brethren in the certainty of medicine itself. Too long, we trust, have we ministered with zeal, and reposed with confidence in that sacred temple, to be now suspected as guilty of a wish to shake its supporting pillars: our desire is merely to call the attention of the profession from the pursuit, or expectation, of new or specific remedies in this disease. The most extended experience has only proved to us that it is best treated by the common means that were familiar to antiquity, and according to the general principles that guide us in the management of ordinary diseases; and we fear that a still more extended experience will only more and more convince us of the comparative inefficacy of our very best treatment,—and more and more impress us with the paramount value of preventive measures—*if, indeed, any such exist!*

Entertaining these sentiments, we need scarcely say, that the announcement of a publication, bearing the title prefixed to this article, excited our liveliest interest—more especially as long experience had taught us, in the treatment of this disease, to trust more to the "Influence of Situation," than to medicine; and as it had been from inquiries directed to this very subject that any hopes we had ventured to entertain respecting its individual or general retardation, had taken their origin. And we must say that, though the work of Mr. M. has fallen far short of what, as philanthropists, we wish, it has scarcely disappointed the expectations which, as physiologists, we had ventured to entertain.—What is the actual amount of this modicum of praise will appear more distinctly in the sequel.

Although Mr. Mansford pretends to considerable novelty in some of the doctrines unfolded in this volume, the paucity of the data on which they are founded enables us to give, in a very small compass, all that is material in its contents. We shall do this, then, in the first place, and uninterruptedly; reserving for the end, any remarks that may suggest themselves during our usual process of analytical condensation.

The volume is divided into two parts: "On the Influence of Situation on Consumption," and "On the Influence of Situation on the Duration of Life." *The first* is occupied in an attempt to prove, as well by reasoning as from statistical documents, that consumption prevails in a degree of frequency proportionate to the elevation of the locality above the level of the sea; or, at least, prevails, much more in high than in low situations: *the second,* that the general duration of life, or longevity, is, also, proportionate to the elevation of the situation. At first sight, these two positions, it must be confessed, present something to our apprehensions not a little contradictory; yet they are rendered sufficiently congruous by the intervention of the author's theory, and are, at least, countenanced by the facts he has adduced.

"Whether an accelerated circulation (says Mr. Mansford) be considered as the cause or effect of the diseased actions which give rise to pulmonary consumption, it is not necessary to inquire. It is sufficient to know, that such a state of the circulation is incompatible with any amendment in the diseased state of the lungs: and the first and most important indication in the curative means to be employed, is to remove or to retard this morbid acceleration, especially in the lungs; and it appears that such a state must be considerably influenced by increased or diminished atmospheric pressure, and that by keeping this fact in view, in our choice of local situation for patients thus circumstanced, we possess additional means for furthering the indication required." P. 15.

"The sensations experienced by æronauts, and by those who have ascended high mountains, may throw some light on this subject. These sensations have been described as occasionally resembling those of intoxication: at others, great lightness and agility have been experienced, but soon terminating in weariness; hæmorrhages from the nose and lungs; sickness; difficulty of breathing, and hurried respiration on the slightest exertion; a quickened pulse; painful distention of the eyes and ears, and palpitation of the heart." P. 16.

Analogous effects, he observes, are produced by submitting "animals and parts of animals," to the action of the air pump; and he conceives the aggravation of symptoms experienced by asthmatics in particular situations, to be often caused by unsuspected superior elevation.

"I found in my own person, (he adds) that an elevation of five hundred feet has caused an acceleration in the pulse of five or six beats in a minute. I have also found, that any motion of the body, as rising from a sitting to an erect posture for instance, has produced a greater corresponding increase of pulsations in the high situation than the low one." P. 20.

If this be the case in health, Mr. M. argues that, in disease of the lungs, "the action of the vessels will be increased in a ratio greatly exceeding that of the other vessels of the body," and that, consequently, "a patient thus circumstanced will experience oppression at the chest, and difficulty of breathing, from the greater influx of blood into the pulmonary vessels." This is proved, Mr. M. conceives, by the effects produced on some consumptive and asthmatic patients, under his care, at a period of a sudden fall of the barometer of an inch and a half.

"They were all worse; they all complained of increased tightness on the chest, and difficulty of breathing; and the pulse in all was quickened. One of these, who had nearly recovered from a long continued and most severe attack of spasmodic asthma, suddenly relapsed." P. 23.

"Another asthmatic, without a regular paroxysm of the disease, experienced great tightness and pain of the chest; and by subsequent inquiries, I found that many others had been similarly affected. One patient emphatically described his sensations, by saying, that he felt as if his body would burst. The effects of this great diminution of atmospheric pressure and its subsequent increase, on the pulse of three patients in different stages of pulmonary consumption, taken at the same hour each morning, were as follow:

| Dec. 8.—Barometer 28.3 | Dec. 9.—Barometer 29.1 |
|---|---|
| M. K. ætat. 24, pulse 116. | ——— pulse 104. |
| S. H. —— 20, —— 116. | ——— 108. |
| J. H. —— 15. —— 120. | ——— 104." P. 24. |

"Two patients with nasal polypi, were also considerably affected. In one case, that of a female, who had not found them very troublesome in general; they descended so as completely to obstruct the passage of the air: but on the following morning, I found that they had resumed their ordinary state. The effects of changes of the weather upon polypus tumours, is experienced by every person troubled with them; but it has usually been ascribed to the relaxing effect of moisture, and partly no doubt with truth; but on the above occasion, the atmosphere shewed no unusual degree of moisture, and was remarkably clear. This expansion of the polypus may serve to show what was going on in the lungs of the asthmatic and consumptive patients; and we shall see that the increase in the number of pulsations, will not indicate the whole of the mischief. For although the lungs contained within their bony case, have no power to increase their volume; the expansion of the blood-vessels will diminish the capacity of the air cells, and satisfactorily account for the aggravation of the symptoms; especially the tightness and difficulty of breathing." P. 25-6.

Mr. M. then proceeds to show, from a topographical survey of the county of Somerset, and medico-statistical documents, "that phthisis pulmonalis is frequent in its more elevated parts, while low and neighbouring lands are comparatively exempt from it." The general results of this investigation are given by the author in the following Table;

TABLE.

| | PLACE. | Population. | Mean Elevation in Feet. | No. of Deaths from Consumption in 1 Year. | Proportion to 1,000 of the living. |
|---|---|---|---|---|---|
| HIGH. | Frome ......... | 10,000 | 450 | 31 | 3.10 |
| | Leigh upon Mendip | 750 | 790 | 3 | 4 |
| | Hinton ......... | 600 | 580 | 2 | 3.36 |
| | Maiden Bradley .. | 500 | 750 | 3 | 6 |
| | Horningsham .... | 1,000 | 650 | 3 | 3 |
| | Kilmington ...... | 600 | 825 | 3 | 5 |
| LOW. | Wells ......... | 5,000 | 50 | 11 | 2.20 |
| | Glastonbury ..... | 4,000 | 25 | 7 | 1.75 |
| | Wedmore ....... | 3,000 | 50 | 4.50 | 1.50 |
| | Axbridge ....... | 1,100 | 50 | — | 0.20 |
| | Wimborne in Dorsetshire ..... | 3,300 | 50 | 7 | 1.95 |

A few pages further on, however, we find a most important modification of this general principle of the beneficial influence of low situations on consumption; nothing less, in short, than that *the neighbourhood of the sea completely does away with the whole of this beneficial influence,* and subjects the patient to equal danger as if he were perched on the very pinnacle,

> Where Andes, giant of the western star,
> Looks from his throne of clouds o'er half the world!—

As illustrating this latter position, he adduces, or rather hints, at the medical history of the East Coast of America,—Carlscrona in Sweden, and Plymouth and Poole in England: and, as a general corollary to his reasonings and inquiries, finally concludes, that "a low inland situation is by far the most favourable for consumptive patients;"—from which axiom naturally flow the objections and recommendations stated in the beginning of this article.

In a work professing to treat of the influence of situation on consumption, the author could not, of course, entirely overlook those situations whose most characteristic quality is an increased and more equable *temperature.* But, although he admits that "the advantages of a mild and regulated temperature, in every stage of pulmonary consumption, are fully established by the practice of some eminent British physicians;" and that "it is frequently observed that phthisical symptoms, contracted in the winter season in this climate, are suspended during the warmer months;—he, nevertheless, unequivocally condemns the practice of sending consumptive patients to warmer climates. And he does so either because the places in these climates usually resorted to, are obnoxious to one or other of the deleterious influences detailed above, (in proof of which he states that phthisis is a frequent indigenous disease;) or on the ground that such practice has been found useless, or worse than useless. He notices the alleged infrequency of consumption in Russia, Sweden, Finland, Denmark, Egypt, Bengal;—and urges the low level of these countries as the probable cause of this happy immunity.

Having thus laid before our readers almost all the facts, theory, and opinions contained in the first Part of Mr. Mansford's Essay, we shall, as usual, submit to them, also, some remarks thereupon that have suggested themselves during the transcription.

In the first place, we must observe, as to the *facts* on which he has grounded his opinions, that his observations have been infinitely too few and confined, as well in respect of time as place, to justify the general conclusions which he has deduced from them. All that we can concede to him, as being fully made out by the documents submitted to our inspection, is—that, *in the year* 1817, *fewer patients died consumptive in the lower parts of Somersetshire than in the higher!* The information he has collected respecting other places, are much too scanty and uncertain to warrant any general conclusions. In this point of view, indeed, we cannot give our author credit either for industry or research. Of the degree of prevalence of consumption in the mountains of Wales and Scotland, on the Alps, Appenines, Pyrenees—or, in short, on any of the mountain-chains in the old or new world, he says not a word! Yet surely some information of this kind, respecting all, or most of these countries, was to be gleaned from the works of foreign travellers, or from the medical and topographical productions of native authors. From this important omission of Mr. M. we do not, however, argue that his opinions are not generally correct; but we must at least doubt of this till we find more evidence adduced in their support. We are afraid, also, that the experience of a single year is much too limited to be safely admitted as a fair specimen of the general prevalence of any disease. And we would earnestly recommend Mr. M. not only to set himself to collect the information above alluded to, but to continue, on an extended scale, his statistical observations in his own neighbourhood.

Tables formed from the results of so short a period are indeed liable to very many causes of fallacy, especially when the materials are so limited as in the case of many of Mr. M's. To notice one only :—Consumption is certainly, in a great proportion of cases, a hereditary disease. Now, suppose a consumptive family, from any other part of the kingdom, had formerly settled in any of the small places noticed by Mr. M. (Maiden Bradley, for example, which has a population of only 500;)—the number of deaths annually occurring among the contaminating progeny, it is obvious, might equal the amount given by him as flowing from the exclusive influence of local situation. But, indeed, the scantiness of his data renders his conclusions obnoxious to more objections than we have room in this place to state.

In respect of the general *principle* on which all the reasoning Mr. M. is founded, and from which he conceives all his alleged facts to flow as necessary consequences,—we may remark, that, although it may be true that aëronauts, and persons ascending high mountains,* and in general, all such as are subjected to the influence of *a recently diminished atmospheric pressure*,—may suffer such derangements in the pulmonary vessels as he describes; we are by no means sure that it therefore follows, that *a residence* in a situation possessing an atmosphere of this character, necessarily entails a greater share of formal pulmonary disease. Does not the undoubting admission of this consequence set undue bounds to the *vis conservatrix*, and plastic energies of Nature? And our author

---

* Mr. M. is probably not ignorant of the opinion of some writers, that, in the case of the *climbers of mountains*, the pulmonary derangements are attributable rather to the greut corporeal exertions used, than to the lessened atmospheric pressure. *See Rees's Cyclopædia.*—Rev.

himself seems inadvertently to have admitted the force of similar reasoning in another part of his work:—

"The functions of life, (he observes) in a state of health, quickly adapt themselves to the exigencies of every occasion, and in the present case the vital power will soon measure its exertions by the scale of resistance, and both regain an exact equilibrium. It does not appear, (he continues) that the pulse of the mountaineer beats any quicker, or that the respiration is more hurried than the inhabitant of the plain; but the heart has much less labour to perform to maintain the very same actions." p. 104.

Now, if the action and resistance are in exact equilibrium, what difference, we ask, makes it whether this effect arises from diminished resistance of the pulmonary vessels and lessened action of the heart,—or from the reverse? But even admitting, to the extent required by our author, the influence of very great elevations, we confess that we feel (independently of reasoning) a great reluctance in yielding our assent to the doctrine that ascribes such effects to varieties of surface of only a few hundred feet. Such an admission, assuredly, seems to us to be yielding too much homage to theory, and too little to the all-perfect wisdom that formed, with capacities of mutual adaptation, "this goodly earth, and all that it inherit." How will our Author's doctrines square with the well-established fact of the coincidence (not to use a stronger expression) of the scrophulous habit and consumption? Is scrophula, also, more prevalent in elevated situations? Or is the alleged coincidence accidental and fallacious.

Among the imperfections of Mr. Mansford's work, we must reckon the total omission of any notice of the general endemial diseases of elevated and Alpine regions. By this oversight, he has certainly deprived himself of data that must have strongly tended to the eduction of the truth, at least, if not to the support of his argument. He has alluded to the "sudden diminution of the weight of the atmosphere," ranked by Dr. Cullen as one of the exciting causes of hæmoptysis; but he takes no notice whatever of the relative prevalence of this disease in Alpine regions: If his theory be true, it ought to be of much more frequent occurrence there, than among the inhabitants of the plain. The same remark applies to several other diseases of the lungs, and also of other parts of the body, which, in congruity with his doctrines, ought to prevail equally with consumption; but he has strangely neglected to take them into account in the statistical reports of the localities he has given.

With regard to another position of our author, viz. ***the malign influence of the sea air,*** or residence near the sea, on consumption,—we confess, that we are even more sceptical than of the truth of the one we have now been considering: and we are the more so, we will confess, that it is so extremely contradictory to that very position. If there be any truth in that opinion, (which, be it remembered, his book is written to support;) surely the admission of another directly the reverse, requires some countenance either from fact or theory. Now, we must say, that Mr. M. has scarcely given us either the one or the other. All the *facts* he has adduced in its support, are contained in the following sentence;

"On the Eastern Coast of America, consumption appears to be nearly as fatal as in Great Britain, while it is much less so in the interior;* it is more prevalent at Carlscrona than in the interior; more so at Plymouth and Poole, than at some distance from the coast."

And on this scanty collection of facts, he has not hesitated blodly to assert the deleterious influence of all situations in the neighbourhood of the sea, however adviseable they may appear to be, on other grounds, as a residence for the consumptive! It is, indeed, not a little surprising, that, in a country, surrounded by the ocean, thickly peopled throughout the whole line of its extensive coasts, and bearing, on its bays and creeks, such a multitude of populous cities, towns, and villages,—*two* places only should have been selected to prove this dogma of the unhealthiness of a sea-side residence. Surely it behoved Mr. M. to have taken a little more trouble to substantiate the truth of so very important a statement,—a statement, moreover, the truth or falsehood of which might have been so very easily ascertained.

Although in possession of documents that tend, as far as they go, to conclusions very different from those of Mr. M. on this particular point, we do not mean positively to say, that his opinion is false; all that we assert is, that he has favoured us with no facts sufficient to justify its adoption. We have, in this disease, so often seen, or fancied that we have seen, so much benefit derived

---

* A Medical Gentleman, of the United States, informs us, that on those parts of the coast, where phthisis is most prevalent, the original Settlers were principally English, as in New England; and queries whether the greater prevalence of the disease is owing to hereditary predisposition. Editor.

from a residence, which, according to his doctrine, ought to be injurious,—that proofs must be numerous and strong indeed, to induce us to admit the hostile influence of sea air, especially when combined with what we still hold to be the most important quality in all residences for such invalids—*an equiable and mild temperature.* This notion of Mr. M. is certainly countenanced by the opinions of several authors (of whose authority however he has not availed himself,) as for example, Sir L. Pepys, Drs. Rush, C. Smyth, Knox, and Irvine; but none of these have adduced facts of any importance in support of their opinions. In the opposite scale we may place (if, indeed, mere authority without corresponding facts were any thing) the opinions of all those authors, antient and modern, who recommend sea voyages. Among these we shall only notice Gilchrist, who not only maintains that the sea air is *beneficial* in this disease, but that consumptions are decidedly *more rare* on the coast than in the interior a few miles distant. In this opinion we are as little disposed to coincide as in the opposite one of our author; although it is one that might claim the countenance of more and greater names than its rival. Mr. M. avoids entering on the *modus operandi* of the sea air in producing its bad effects; but, speaking of Madeira, he incidentally mentions, as one of the causes of its ineligibility, "the *too stimulating effects* of a marine atmosphere." What is intended to be conveyed by this expression we profess not precisely to comprehend: as a counterpoise to its theoretical value (whatever it may be) we shall simply notice *the fact* stated by Dr. Trotter, of the infrequency of consumption among the very men "whose home is on the main."

According to this doctrine not only *seamen*, but the inhabitants of *all small islands*, and all *fishermen*, ought to be more subject to phthisical complaints than other people. Is this really the case? If the alleged evil influence of littoral residence arises from marine exhalations,—what ought to be the state of pulmonary diseases among the native fishermen of Newfoundland, who live in a perpetual fog or mist, the produce of the ocean? As illustrating our author's notions of the rationale of the deleterious influence in question, as well as affording a specimen of the reasoning he occasionally has recourse to, we may notice the following "remarkable fact."—Finding the proportion of deaths from consumption in Carlscrona too low, according to the exigencies of his theory, for a sea-side locality, he considers it in reference to this, "worthy of observation, that the waters of the Baltic contain but a little more than one-fifth of the saline matter contained in the Atlantic!!" With equal sanction from philosophic reasoning might he seek for the cause of this comparative immunity of the natives in the structure of their language or the fashion of their doublets! Not that we positively mean to assert that even *this* idea of our author's is totally unfounded,—but only that his entire want of investigation on this head renders any such speculation perfectly inadmissible. Would that the forthcoming "*Medical Logic*" of Sir Gilbert Blane might banish from our science this eternal disgrace and bane —the *post hoc ergo propter hoc* mode of reasoning!

We have dwelt the more particularly on this point because the alleged fact, if true, must not merely overthrow opinions which we have long entertained, (which, it must be allowed, would be but a very trifling evil) but, if admitted as such by the profession, may tend materially to counteract a mode of proceeding, which, we are convinced as well by reason as by experience, is the most important in this most important disease;—we allude to the practice of sending consumptive patients to the southern parts of our own island, or to the still milder shores of the Mediterranean. On this subject we beg leave to quote the words of a respected colleague; with which, both as pointing out what appears to us to be the *true indication* of cure, and as stating a fact most consentaneous to our own practical experience in the history of this disease,—we most readily and entirely accord.

"As the predisponent, or what may be termed the hereditary causes of consumption are beyond our controul, we can only look to those which usually excite the disposition into action; and as the state of the atmosphere has been shown to influence scrofula, and, in general, to produce catarrhal complaints, so our principal object must be directed to the regulation of this important agent.*

---

* A remarkable and most striking illustration of the influence of a cold climate to excite phthisis, and of the preventive powers of a mild one, is given in the last number of this Journal, (p. 259) from the extended experience of a correct observer, M. Broussais.

The same comparative influence of hot and cold climates in exciting pulmonic disease, may (as a medical friend lately observed to us) be seen at once by any person that attends public worship, even for a single Sunday, in England and between the Tropics: in the former country, especially in winter, *coughing*, in all its varieties and degrees, is never unheard, and too often literally "drowns the parson's saw;" while, in the latter, it is almost entirely unperceived. Rev.

We must take care, however, in sedulously guarding against colds not to render ourselves enervated exotics by heat. The only effectual means of resisting any injurious impression to which we are unavoidably exposed at times, is by careful, gradual exposure to its operation. By attention to dress, however, we can defend ourselves, in a great measure, from sudden vicissitudes, particularly from heat to cold; and, where circumstances will admit, a removal to a mild and warm climate, will obviate the necessity of being ever on the watch. A tropical climate is not the safest asylum for the pulmonary invalid. There the transitions of temperature are often rapid, and the lungs are apt to participate with diseases to which other organs, particularly the liver, are peculiarly prone beneath the torrid zone. In the warmer countries of Europe, though the houses are, in general well adapted towards obviating atmospherical heat; yet from their construction, the pulmonic invalid is there peculiarly liable to catch cold; so that, perhaps, little advantage is gained, on the whole, by emigration abroad. It is certain, however, that *pure* air and *elevated situations* are prejudicial. The more equable temperature *of the sea* has *long been known as favourable to phthisical patients*. The southern and western counties of England and Wales afford secluded vallies where the bleak north-east wind can hardly penetrate, and where the surface of the earth is not long bound in frost. There a limited indulgence in gentle horse exercise, by visiting the neighbouring *shores* in fine weather; guarding against every stimulus in diet; and yet admitting a small portion of light animal food, with moderate warm dress, will often prevent the predisposition from coming into action for a long time—perhaps till the phthisical period has elapsed altogether."*

In the above paragraph is contained, we are sorry to say, *almost* all that the experience of ages has produced as most useful in the treatment of this disease; and when to what is there noticed we add the employment of the ordinary means, for reducing inflammation when it arrives at any height, and for the relief of other troublesome symptoms as they arise, we fear we have stated *all!*

On the subject of *temperature,* as a quality to be considered in the choice of a situation for the victim of pulmonary disease, our author is extremely brief and unsatisfactory. He furnishes us with not one new fact or observation, and makes but little use of the vast store of materials collected on this subject by the many authors that have preceded him. Indeed we are of opinion that Mr. M. would have more consulted his own reputation had he confined himself exclusively to the consideration of

* Dr. Johnson on the Atmosphere, 1st edition, page 22.

the effect of elevation on this disease, as, it is obvious, this is the only part of his subject that he has studied with any sort of attention.

As a conclusive argument against the practice of sending "consumptive patients to seek relief in southern latitudes," he contents himself with referring his readers to "the cemeteries of Lisbon, Nice, and Madeira,"—which, without directly saying so, he wishes them to imagine as crowded with the remains of the expatriated and deluded victims of this disease! Now, we must take the liberty of saying, that this is a mode of reasoning most unworthy the liberal cultivation of science; as it too evidently appears to us to be purposely fitted for "the lowest capacity." Surely Mr. M. is not ignorant of the condition of the great majority of consumptive patients that go abroad in search of health; and requires not to be told that the death of thousands of invalids, that may reach any particular spot at an incurable stage of the complaint, is no argument against the beneficial influence of that spot in an earlier period of the disease, when only it is either curable or susceptible of retardation. He tells us in his Preface, that his book is intended for the general reader as well as the professional: we will not say so harsh a thing as that the mode of stating the above circumstance was *intended* to mislead the former; but we feel assured that it is admirably calculated to do so. If the author is not aware of this, he must be simple indeed; if he is, we will spare him the epithet his wilfulness deserves.

The other argument adduced against the practice of sending consumptive patients abroad,—viz. the alleged prevalence of phthisis in these countries as an indigenous disease,—although, perhaps, in the present state of our knowledge, a sufficiently safe criterion to go by,—is, nevertheless, we conceive, not entirely free from objection. May not a climate possess some particular qualities that render it eligible as a residence for invalids,—even although that very disease, for the relief of which it is sought, be of frequent occurrence there? If, as our author admits, "the advantages of a mild and regulated temperature, in every stage of pulmonary consumption, are fully established,—does the mere existence of this disease in climes enjoying this very quality, necessarily destroy its known beneficial effects? Are we not rather intitled to conclude that the disease in these countries springs from *other* qualities, which they possess in common with ruder climes, and that their character of mildness and equability of temperature, must still exert a beneficial influence, not only on the exotic, but the indigenous affection? Although not sufficiently potent to prevent the operation of the deleterious causes, it may yet have power to check the ravages, and thus, at least, retard the progress of the disease.*

The principal objections, we conceive, to a residence abroad, arise, not from the inutility of a milder climate in consumption, but because the acquisition of this desirable object is attended, in a great many cases, by many and great inconveniences. Among these, we may notice the decidedly injurious effects of the high summer heats in Portugal, Spain, France, Italy, and Malta; the great variableness of temperature, even in winter, in the former; the uncomfortable domestic accommodation in all of them; not to mention the separation from friends and the loss of an English home, at a period when the presence of friends and the comforts of home are most wanted. To this last class of inconveniences only, Madeira appears to us to be obnoxious, at least in the early stages of the complaint; in the latter stages, we fear that neither it nor any other place possesses, all things considered, any advantages sufficient to allure the devoted victim from his native land!

Upon the whole, although that part of Mr. Mansford's Essay, that relates to the injurious effects of an elevated locality in cases of consumption, seems borne out by some reasoning, and even countenanced by some facts,—certainly not contradicted by any experience of our own,—we must beg leave still to recommeud our patients to seek for relief in the "myrtle vales" of our south-western counties, even although open to the full influence of the "too stimulating effects of a marine atmosphere;" and we shall continue to advise this "situation," even in preference to the romantic vales so much lauded by Mr. M. as long as it continues to possess a mean winter temperature so very superior to any part of Somersetshire, or indeed any other part of the kingdom;—unless, indeed, Mr. M. shall adduce more reasons than he has yet done, to convince us that the neighbourhood of the sea, which gives it its mildness, more than counter-balances this good quality.

* See the observation of Broussais already referred to.

While on this subject,—although not particularly called for by any thing in the work under review, yet as bearing directly on the subject it proposes to treat,—we beg leave to offer a few observations on the situation that appears to us the most eligible in this country for consumptive patients. *Mildness and equability of temperature* (and it may be *safe*, at least, to add—*a low level*,) we have already stated to be the most essential qualities of the climate suited for such invalids; and of all places in this island we know, from undoubted information, that the south-western part of Cornwall possesses these qualities in the most eminent degree. This superiority in point of mildness has been noticed by many writers; but has more recently been impressed upon *our* minds by the observations of a distinguished member of the college, who has lately returned to town, after a residence of several years in that part of the country.* From the same most respectable source of intelligence, we have reason to believe that the medical world may expect ample details of the peculiar climate of that part of the island, and of its highly beneficial influence in the incipient stages of pulmonary consumption. In the mean time, we beg leave to call the attention of our readers to the following extracts from Dr. Young's Essay on Climates, in his excellent and most elaborate work on Consumptive Diseases:

"It appears from this comparison, that none of the situations here enumerated, north of Lisbon, *except Penzance*, has any material advantage over London, in the mildness of its winter. The best parts of Devonshire seem to be about a degree and a half warmer; Torquay, however, may perhaps be a little milder than this; but Penzance may be fairly considered as having a temperature of four degrees and a half higher in the coldest months; nor are the journals here employed the only ones which allot such a superiority to the climate of this extremity of our island." *Treatise p.* 87.

"It does not appear that Devonshire possesses any decided advantages over London, with respect to *equability* of climate, if we judge of the climate of London from the observations made at the apartments of the Royal Society only; ******* in this respect too Penzance retains its superiority over Devonshire.—*Ib.* p. 92.

We make no apology for this digression from our analytical path. We are ourselves so convinced of the para-

---

* John Ayrton Paris, M. D. F. L. S. of Dover Street, one of the Censors of the College, and author of that very useful little work "Pharmacologia"—of which we are glad to see a new and enlarged edition announced.—Rev.

mount importance, in consumption, of a climate possessing the above qualities, that we are anxious to lose no opportunity of impressing the same on the consideration of our readers; and we, moreover, feel particularly happy, in the present ominous aspect of continental politics, to be able to point out to such as are ignorant of the fact, that we possess, in our own island, more than one situation, that renders us, in the treatment of this direful malady, more independent of foreign countries than is usually imagined.

After the very full consideration we have bestowed on the first part of this Essay, our limits will permit us to offer only a few brief remarks on the second Part.

"As life advances, (says Mr. M.) the vigour of its active principle declines; while mechanical obstructions from various causes, accumulate to oppose its functions, until resistance overbalances power, and life is extinguished. The soft parts grow rigid; the vessels themselves sometimes grow ossified, and their extreme branches obliterated; the valves of the veins frequently relaxed, or ruptured, cease to perform their office, and the tardy column of blood is with difficulty urged on to complete the circulation. Under such accumulated circumstances of oppression, how great must be the labour which the heart has to perform! at a time too when the sinking powers of Nature require a lightened, rather than an augmented load." P. 74.

*A rarified atmosphere*, our author conceives, affords this lightening; and the whole of this part is occupied in an attempt to prove this by the same sort of evidence given in the preceding inquiry. In every climate, he contends,

"The average duration of life corresponds with the elevation of the different countries, or of their respective parts. So universally does this axiom appear to hold good, that the general elevation of a country being given, the probable age of its inhabitants may with tolerable accuracy be ascertained." P. 76.

In proof of this he adduces the longevity of the South American Highlanders, the inhabitants of Caucasus, and the Alps, &c. &c.

"The dangerous age in elevated situations is the consumptive one; that passed, the probabilities of life will be found *not* to have decreased in proportion to the increase of years." P. 93.

As practical corollaries resulting from his inquiries, in this part of his work, he counsels *the convalescent from acute disease*, and *the aged*, to seek, in elevated situations, the former the restoration of health, the latter the prolongation of life.—As, however, he believes that "the duration of life is also proportioned to the temperature of the country or spot inhabited," p. 130,* he finally concludes, "that the fittest residence for the aged and the invalid, is that, where elevation can be united with mildness of temperature." These precious boons he admits are to be obtained in the highest perfection in foreign countries; but even in this case, also, his favourite Somersetshire offers, in her all-sheltering bosom, a blest alternative to those "whom fortune has not gifted with the means of seeking better climates."

"Such may seek shelter in the south-western counties of this island; whose milder air, and bold elevations, afford an infinite variety of situations answering the object desired."

Among these, "the higher parts of Bath and Clifton may be particularised, the former especially;" &c. But we must now take our leave both of Somersetshire and of Mr. Mansford. That he has established his theory we by no means allow; that he has succeeded in proving his native county to be the best station for consumptive patients, we are still further from conceding; we have found cause, more than once, to impugn his character for industry and research; and he can have little claim to ingenious or profound physiological reasoning: notwithstanding all this, we are still of opinion, that,—as he has added some facts to the existing stock, and has called the attention of medical men to the more particular consideration of a cause of disease, which, possibly, may be more important than has hitherto been suspected,—he is well entitled to the thanks of the profession; and if he will only condescend to follow the honest counsel we have ventured to offer,—we doubt not, that he will yet be able to present the public with a work more worthy of its notice and his own talents, and more calculated to add to his permanent reputation.

---

* What will the cemetries of the inter-tropical regions say to this? Rev.

